# Dynamics of a bottom-heavy Janus particle near a wall under shear flow†

**DOI:** 10.1039/d5sm00229j

**Published:** 2025-06-18

**Authors:** Zohreh Jalilvand, Daniele Notarmuzi, Ubaldo M. Córdova-Figueroa, Emanuela Bianchi, Ilona Kretzschmar

**Affiliations:** a Department of Chemical Engineering, City College of New York (CCNY), City University of New York (CUNY) 140th Street & Convent Avenue, New York New York 10031 USA kretzschmar@ccny.cuny.edu; b Institute for Theoretical Physics, Technische Universität Wien Wiedner Hauptstraße 8-10 A-1040 Vienna Austria daniele.notarmuzi@tuwien.ac.at; c Department of Chemical Engineering, University of Puerto Rico-Mayagüez Mayagüez PR 00681 USA; d CNR-ISC, Uos Sapienza Piazzale A. Moro 2 00185 Roma Italy

## Abstract

In this study, Brownian dynamics simulations are implemented to investigate the motion of a bottom-heavy Janus particle near a wall under varying shear flow conditions and at small Péclet (Pe) numbers. The stochastic motion of the Janus particle impacted by surface forces is described using a set of coupled Langevin equations that takes into account the Janus particle orientation. Interactions arising from surface potentials are found to depend on the separation distance between the Janus particle and the wall, the properties of the surfaces involved, and the thickness of the Janus particle cap. When shear flow is introduced in the system, the dynamical behavior of the Janus particle is also governed by the strain rate. Furthermore, the effect of friction on the dynamical behavior of the Janus particle under shear flow is investigated and reveals that the rotational motion of the Janus particle slows down slightly when the particle is close to the surface. In summary, we demonstrate the ability to utilize Brownian dynamics simulations to capture the rich dynamical behavior of a bottom-heavy Janus particle near a wall and under a range of shear flow conditions, cap thicknesses, and surface charges.

## Abstract 2

In this study, Brownian dynamics simulations are implemented to investigate the motion of a bottom-heavy, charged Janus particle near a charged wall under varying shear flow conditions.

## Graphical abstract

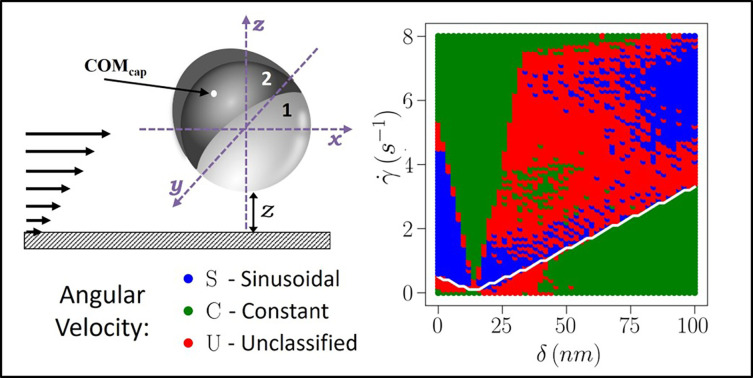

## 1 Introduction

Besides their ubiquity as amphiphilic colloids in nature, Janus colloids have a wide variety of applications in areas such as biomedicine,^1–3^ energy storage,^4^ catalysis,^5^ and environmental science.^6^ In many of these anticipated applications Janus particles will encounter various types of boundaries such as fluid/fluid interfaces^7^ and solid walls of blood vessels, channels, and porous structures. As such, understanding and predicting individual particle–wall interactions is important. For example, particle–wall interactions can determine drug delivery rates,^8^ guide particle swimming,^9–11^ result in scale formation in pipes,^12^ or change the flow in porous media.^13^

There is a long scientific history regarding the description of particle–wall interactions.^14–19^ Most of the literature addresses spheres or ellipsoids near boundaries with a few studies focusing on spherical passive Janus particles discussed below. Experimentally, three techniques, atomic force microscopy (AFM), surface force apparatus (SFA), and total internal reflection microscopy (TIRM), have been used to measure particle–wall interactions. AFM and SFA require the immobilization of the probe particle,^20–22^ whereas TIRM does not. TIRM^23^ is an experimental light scattering technique that indirectly measures particle position above a surface as a function of time resulting in a probability density function (PDF), *p*(*h*), in which *h* is the height of the particle surface from the wall. It then infers the particle–wall potential, *U*(*h*), from analysis of the PDF. The particle–wall interaction potential and scattering morphology become more complex functions when Janus particles are involved due to their non-uniform surface charge and potentially heavier cap that influence particle height and orientation in a complex manner. In a recent perspective article, Yan and Wirth^24^ introduced Scattering Morphology Resolved TIRM (SMR-TIRM) and demonstrated that assessment of anisotropic particle interactions near boundaries with SMR-TIRM is more accurate when combined with Brownian dynamics (BD) simulations. Using BD simulations, Rashidi *et al.*^25,26^ explored the dynamics of a polystyrene Janus particle with a non-uniform surface potential with and without a gold cap in the vicinity of a negatively charged wall and successfully derived the 3D potential energy surface of the particle that can be used in the interpretation of TIRM measurements. In a follow up study, Rajupet *et al.*^27^ used the surface element integration (SEI) method to elucidate the impact of van der Waals forces on Janus particle–wall interactions and found that at short distances (relevant in systems of high ionic strength) van der Waals interactions and cap nonuniformity significantly impact the potential landscape experienced by the Janus particle. More recently, the focus has been on understanding the behavior of Janus particles in channels under shear flow, which further complicates the interpretation of TIRM data. Therefore, the development of robust theoretical models and their numerical analysis are a central tool to fully capture the physics of Janus particles, as models are needed to interpret experimental results and, at the same time, might predict novel behaviors to be in turn verified experimentally.

A number of simulation studies have investigated the dynamic behavior of Janus particle suspensions in channels and near solid boundaries with focus on particle–particle and particle–wall interactions under shear flow. Using BD simulations, Mohammadi *et al.*^28^ investigated the binding kinetics of Janus particles under shear flow and determined the coagulation rate of a particle pair. Kobayashi *et al.*^29^ studied the rheology of Janus nanoparticle suspensions in nanotubes of varying surface chemistry with dissipative particle dynamics and found that flow properties strongly depended on the structure of the colloid phase and the wall chemistry. Nikoubashman *et al.*^30,31^ employed hybrid molecular dynamics simulations to investigate the shear-induced break up of Janus particle micelles within a slit-like channel under shear flow and found that the shear flow can lead to enhancement of micelles and the formation of bigger aggregates some of which show enhanced stability due to their cluster symmetry. In a follow up study,^32^ they found that increasing particle–wall interactions causes adhesion and cluster formation on the channel walls. DeLaCruz-Araujo *et al.*^33^ showed using BD simulations that exposure of micellar, vesicular, worm-like and lamellar Janus particle aggregates to shear flow induces rearrangement, deformation, and break-up of the aggregates that is more easily controlled by the Peclet number (Pe) than the interaction potential. In a second study,^34^ they investigated the switch between parallel and perpendicular alignment of lamellar Janus particle aggregates by shear flow and developed a simple scaling argument based on the torque balance on a single Janus particle pair.

Other studies have focused on a single passive Janus particle near a wall. For example, Ramachandran *et al.*^35^ used a finite element method to study the dynamics and rheology of a dilute suspension of slip-stick Janus particles in creeping flow. They showed that depending on the ratio of the slip length to the particle radius the rotational motion of the particle varies. In addition, the translational motion of the particle is predicted to be impacted by the initial orientation of the particle. Furthermore, the suspension exhibits non-Newtonian behavior for a specific volume fraction of slip-stick Janus spheres. Troufa *et al.*^36^ numerically simulated the dynamics of a slip-stick Janus sphere within a Newtonian fluid confined in a cylindrical micro-channel under Poiseuille flow and found two behavior regimes where the particle either shows periodic oscillations near the wall or migration towards the channel center if near the centerline.

Most recent studies have primarily focused on the steering of active particles near walls,^37–41^ though open questions regarding the interactions of passive Janus particles with walls under shear flow persist. Additionally, Janus particles with thick caps (50–100 nm) have been explored, where the cap volume is required to attain the field interaction strength needed to control the Janus particle behavior.^42–47^ Understanding how the interplay of the particle's and substrate's properties influences the particles height, rotation speed, and the frequency with which its surface interacts with the substrate is essential for advancing applications in self-assembly, catalysis, drug delivery, and biosensing.

Here, a BD model is introduced that captures the interaction of a bottom-heavy Janus particle exhibiting two distinctly charged surfaces with a charged wall with and without shear flow. The BD model is based on the Hogg, Healy and Furstenau (HHF) sphere–sphere interaction model^48^ modified to apply to a sphere-infinite plate interaction by setting one of the sphere radii to infinity. Note, the model is limited to thin double layers (*i.e.*, *κR* ≫ 1). The BD model is used to capture the non-trivial interplay between surface properties of the particle, *i.e.*, surface potential and cap thickness, and the nearby wall in the presence of shear flow, where surface forces and hydrodynamic interactions (using Goldman *et al.*'s dimensionless frictional coefficients)^49^ govern the dynamics and determine the particle height.

## 2 Simulation model

**Fig. 1:**
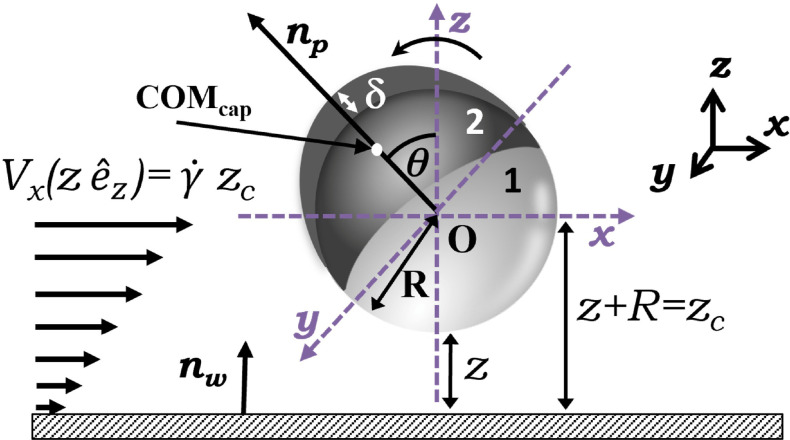
Schematic representation of a bottom-heavy Janus particle capped on face 2 with a metal of thickness *δ* and a cap center of mass COM_cap_, bounded with a wall w and subject to shear flow of strain rate *γ̇*. *θ*, indicates the particle orientation with *θ* = 0 indicating a cap/face 2 up orientation, while *θ* = π indicates a cap/face 2 down orientation. Other relevant parameters are also depicted (see the text).

A hard sphere of radius *R* with two different hemispheres (1,2) serves as a model particle for a system in which each face is uniformly (assuming constant surface potential), but independently charged, creating the Janus particle (Fig. 1). The Janus particle is suspended in an aqueous solution of ionic strength *I*, which throughout this study is considered to be deionized (DI) water (Millipore, resistivity 18.2 MΩ cm and viscosity *μ* = 1 × 10^−3^ Pa s at 25 °C, *I* = 1 × 10^−6^ M). The Janus particle is bounded by a wall, *i.e.*, a substrate made of SiO_2_, which is uniformly charged. The normal to the particle (***n***_p_), that passes through the center of the particle and through the cap's center of mass (COM_cap_) is employed to characterize the rotational behavior of the particle, shown in Fig. 1. Finally, the Janus particle is subjected to a shear flow with varying strain rates *γ̇*.

Anisotropic interactions between the Janus particle and the wall are modeled by utilizing the total interaction energy, *U*_tot_, as the sum of electrostatic double layer (EDL) interactions and the gravitational interaction. Hence, *U*_tot_ is given by eqn (1):^50,51^

1 *U*_tot_(*z*, *θ*, *ψ*_1_, *ψ*_2_, *ψ*_w_) = *U*_EDL_(*z*, *θ*, *ψ*_1_, *ψ*_2_, *ψ*_w_) + *U*_gravity_(*z*),

where *z* denotes the height of the Janus particle, *i.e.*, the distance of the Janus particle from the wall, *θ* is defined as the angle between the normal to the particle (*i.e.*, unit vector ***n***_p_) and the normal to the wall (*i.e.*, unit vector ***n***_w_), and *ψ*_1_, *ψ*_2_, and *ψ*_w_ are the surface potentials of the two faces and the wall, respectively. The contribution from the EDL interaction between a uniformly charged particle and a uniformly charged wall (based on the modified HHF model) is given by eqn (2):2
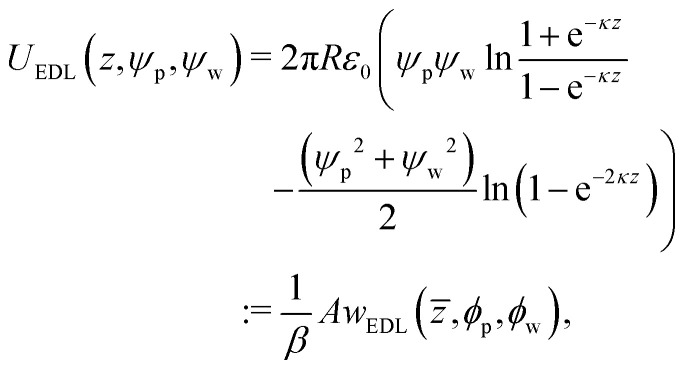
where *A* is introduced as a dimensionless parameter, eqn (3a), and *w*_EDL_(*z̄*, *ϕ*_p_, *ϕ*_w_) as the dimensionless EDL interaction, eqn (3b):

3a *A*: = π*Rε*_0_*ψ*_0_^2^*β*,

3b
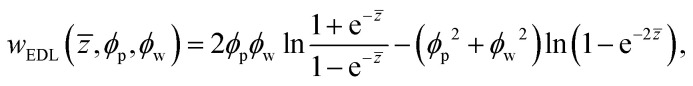
with *z̄* = *κz*, where *z* is the height of the particle non-dimensionalized by measuring the length scale in units of Debye screening length, *κ*^−1^. In eqn (3a), *ψ*_0_ = 1/*qβ* is the thermal electrostatic potential used to non-dimensionalize the wall and surface potentials as *ϕ*_i_ = *ψ*_i_/*ψ*_0_ with i = w and p, respectively, where *q* denotes the electron charge (in absolute value) and *β* = 1/(*k*_b_*T*), where *k*_b_*T* is the thermal energy. Note the same notation is used in the ESI,† Section S1, which provides additional information on the derivation. Use of the modified HHF model for the sphere-infinite plate interaction potential assumes a small constant surface potential (<25 mV), *i.e.*, the Debye Hückel approximation, and small double layer thickness compared to the particle size, *i.e.*, the Derjaguin approximation. HHF showed in their work that the model can be extended to surface potentials of 50–60 mV.^48^

The Janus particle assumes various configurations in proximity to the wall and each hemisphere of the Janus particle contributes its own particle–wall EDL interaction to *U*_EDL_. The anisotropic interaction is a function of the Janus particle orientation with respect to the wall as specified by *θ* (see Fig. 1). The orientation of the cap is incorporated into the model using the orientation-dependent factors 
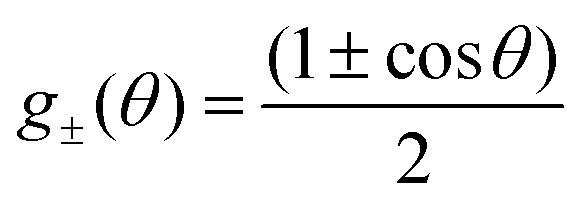
 yielding *U*_tot_ for a Janus particle interacting with a wall, eqn (4):4
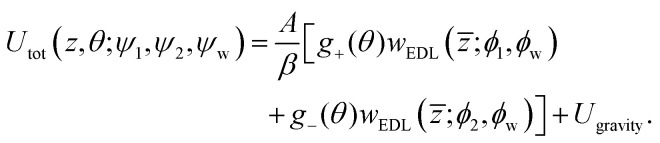


The governing equations that describe both the translational and rotational motion of the Janus particle near the wall, known as Langevin equations, are:

• Translation of particle center, O, along the *x* direction:5
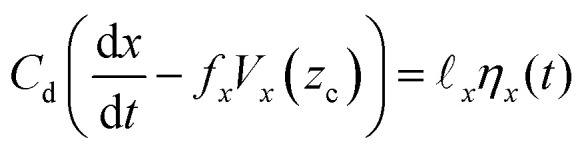


• Translation along the *z* direction:6
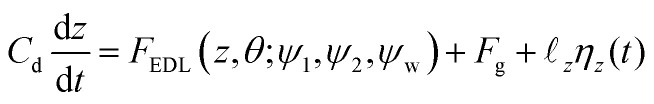


• Rotation around the *y* axis (passing through particle center, O):7
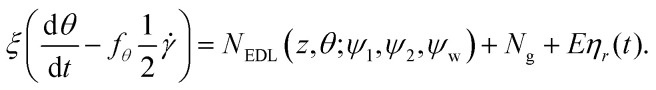


In the above equations, *C*_d_ = 6π*μR* and *ξ* = 8π*μR*^3^ are the translational and rotational drag coefficients, respectively, and *f*_*x*_ and *f*_*θ*_ are Goldman *et al.*'s^49^ dimensionless frictional coefficients for translational and rotational motion. Note that expressions for *C*_d_ and *ξ* represent a perfect sphere and that the contribution from the cap to the particle shape is neglected here. *V*_*x*_(*z*_c_) = *γ̇z*_c_ denotes the velocity of the external shear flow that is being exerted on the position of the center of the Janus particle, O (see Fig. 1), by the externally applied strain rate, *γ̇*. ℓ_*x*_*η*_*x*_(*t*) and *ℓ*_*z*_*η*_*z*_(*t*) are the Brownian forces in the *x* and *z* directions, respectively, while *Eη*_*r*_(*t*) is the rotational Brownian force. *F*_g_ = m*g and *N*_g_ = −Ξ*sin(*θ*) are the gravitational force and torque, respectively, where m* and Ξ* correspond to the effective mass of the particle and effective weight of the cap, respectively. Note that in the absence of shear flow, *γ̇* = 0, one clearly has *V*_*x*_ = 0. Finally, *F*_EDL_ and *N*_EDL_ are the contributions from the electric double layer interaction and defined as:8
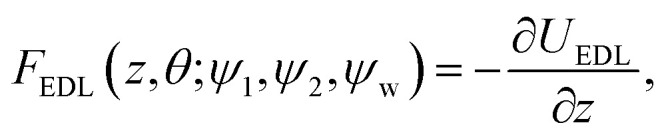
9
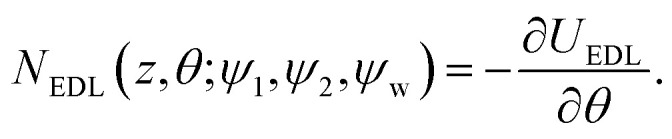


The conservative wall–particle interaction forces are computed through the wall–particle interaction energy as *F*_tot_ = −∇*U*_tot_, which includes the electrostatic double layer interaction and gravitational potential energy. Eqn (5)–(9) describe the most general system under analysis in this work and is referred to as model IV. In particular, they account for different surface potentials of the two sides of the Janus particle, an asymmetry in the mass distribution as implied by the cap of thickness *δ*, the torque induced by the shear flow, and the hydrodynamic friction. Note that an imbalance in the surface potentials and the mass asymmetry induce a force, *F*_EDL_ and *F*_g_, respectively, as well as a torque, *N*_EDL_ and *N*_g_, with the forces acting also on symmetric systems while the torques are zero.

Before considering model IV, we first consider two simpler models of Janus particles that assess the role of various parameters appearing in the governing equations (eqn (5)–(9)). In Section 4, results from model 0 and model I are presented. Model 0 is not truly a Janus particle: the cap is absent (*δ* = 0, homogeneous mass distribution) and the surface potential of the two faces is identical (*i.e.*, *ψ*_1_ = *ψ*_2_), making it impossible to distinguish between them. Model I differentiates the role played by inhomogeneity in the surface potentials (*ψ*_1_ ≠ *ψ*_2_) of the particle and is the simplest representation of a Janus particle considered in this work. Note that both model 0 and model I represent particle behavior in the absence of shear flow, *i.e.*, *γ̇* = 0 and therefore *V*_*x*_ = 0. In Section 5 the cap thickness is introduced, *i.e.*, we consider *δ* > 0, while still keeping *γ̇* = 0 (hence *V*_*x*_ = 0) and we refer to this formulation as model II. In model II as well as in the subsequent models III and IV, where *δ* > 0, the geometry of the cap is assumed to be thickest at the pole and becomes gradually thinner as it approaches the equator of the particle, which agrees well with recent experimental observations.^52^ The cap volume is estimated as the volume of a cylinder with a diameter of 2*R* of thickness *δ*, *V*_cap_ = π*R*^2^*δ*. Typically, Janus particles are coated with a thin layer of metal, *e.g.*, platinum, which is denser than the SiO_2_ core of the particle (*e.g.*, *ρ*_Pt_ = 21.45 *vs. ρ*_SiO_2__ = 2.64 g cm^−3^). Consequently, both the density mismatch and the geometry of the cap render the particle “bottom-heavy”. Model III differs from model II as the effect of shear flow (*i.e.*, *γ̇* ≠ 0) is included in the Langevin equation through a non-zero velocity term, *V*_*x*_. The system Pe number at the highest shear flows studied here (*γ̇* = 8 s^−1^) is small (Pe < 0.006) using the Debye length (*κ*^−1^) as the geometric length scale. Note even when the particle diameter is used as the geometric length scale, the system Pe < 1. For the particle, the ratio of the advection to Brownian forces for the particle is expressed by the particle Péclet number, Pe_p_ = *γ̇R*^2^/*D*_0_, where *D*_0_ is the translational diffusivity of the isolated Brownian particle in the bulk (ESI,† Section S1). Depending on the particle size and strain rate, Pe_p_ numbers vary over the range of 0 ≤ Pe_p_ ≤ 0.5 × 10^4^. Subsequently, the effect of the hydrodynamic friction is evaluated by inclusion of frictional parameters, *f*_*x*_ and *f*_*θ*_, in the translational and rotational terms in model IV. Table 1 summarizes the model parameters for radius (*R*), surface/wall potentials (*ψ*_1_, *ψ*_2_, and *ψ*_w_), cap thickness (*δ*), and shear rate (*γ̇*).

**Table 1:** Parameters used in this study

Parameter	Symbol	Value
Particle radius	*R*	0.75, 1, 2, 4 μm
Potential on face 1	*ψ*_1_	−20, −40 mV
Potential on face 2	*ψ*_2_	−20, −40 mV
Wall potential	*ψ*_w_	−20, −50 mV
Cap thickness	*δ*	0–100 nm
Shear rate	*γ̇*	0–8 s^−1^

Note that, in all equations the particle height (*z*) and position (*x*), time (*t*), force (*F*), and the torque (*N*) are non-dimensionalized by the Debye length (*κ*^−1^), the characteristic time 
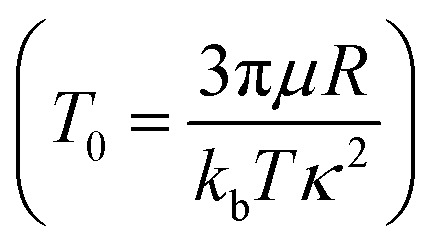
, thermal force (*k*_b_*T*/*κ*^−1^), and thermal energy (*k*_b_*T*), respectively. Non-dimensionalized variables are indicated by a bar, *e.g.*, *z̄*.

## 3 Methods

A BD simulation is conducted to evaluate the rotational and translational trajectories of a Janus particle by integrating the Langevin equations forward in time employing the explicit Euler time integration scheme. Thermal fluctuations are incorporated in the integration by sampling from the normal distribution *N*(0,1). Note the standard white noise assumption for the Brownian force applies for the system studied here as the ratio of *γ̇* over the bath frequency (≈10^12^ s^−1^) is less than 10^−11^. For larger ratios, *i.e.*, very large *γ̇* or more viscous fluids, a non-white noise more accurately describes the random fluctuations.^53^ Each state point is integrated from time *t*_0_ up to time *t*_1_ and *N*_p_ independent particles are simulated. Specific settings of numerical simulations are detailed in Section S2 of the ESI.†

Analysis of the trajectories results in PDFs that show the frequency with which the particle is found in a particular orientation and height at various conditions. In order to assess and minimize bias in the behavior of the Janus particle with respect to the initial position and orientation, random initial positions and orientations are utilized. Equilibrium behavior of the Janus particle is verified to be independent of the initial condition and the early-time stages of the dynamics are disregarded from the data analyzed, removing data from the beginning of the simulation up to the equilibration time *t*_eq_ when necessary. See the ESI,† Section S2 for details on the initial condition and on the equilibration time. This approach ensures that the results obtained studying different trajectories of the same Janus particle are statistically equivalent.

When the shear flow is included in the model, *i.e.*, model III, the focus is particularly on the rotational degree of freedom of the Janus particle. To characterize this, the average angular velocity of each trajectory is measured by temporally averaging the velocity as10
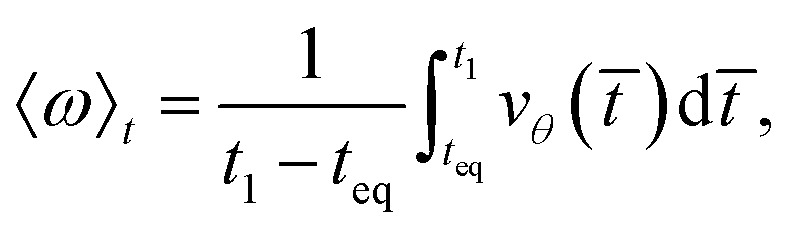
where the instantaneous angular velocity *v*_*θ*_(*t̄*) is simply estimated as (*θ*(*t̄* + d*t̄*) − *θ*(*t̄*))/d*t̄*. The ensemble average of the angular velocity is then computed by averaging over different simulations, obtaining a constant angular velocity value, *ω*. The *ω* value is used as the order parameter of a dynamical transition between a rotating state, where *ω* > *ω*_0_, and a non-rotating state, where *ω* ≤ *ω*_0_. The two states are distinguished by setting the threshold *ω*_0_ = 0.05 and verifying the robustness of the results by considering similar values of the threshold.

The trajectories, *θ*(*t̄*), exhibit a rich behavior, which is classified using statistical testing. Specifically, time is binned and an average value of the instantaneous velocity *v*_*θ*_(*t̄*) is computed for each bin to obtain a time-dependent average angular velocity *ω*(*t̄*) (see the ESI,† Section S2), whose behavior is fitted to two functions: a constant and a sinusoidal. A constant behavior of *ω*(*t̄*) indicates that the particle rotates with a velocity that is independent of its orientation and possibly does not rotate at all, if *ω* = 0; a sinusoidal behavior indicates that the rotation is accelerated over time, an effect that is the consequence of the complex interplay between shear forces, gravitational attraction between the particle and the wall and electrostatic repulsion between the wall and the two sides of the Janus particle (which in general have different surface potentials and hence experience different repulsion strengths). The best model among the constant and the sinusoidal is selected using the Akaike Information Criterion,^54^ which appropriately accounts for the larger number of fitting parameters (four) that the sinusoidal model has over the constant (which has one). In this way, each trajectory is classified as being sinusoidal or constant, providing a number of classifications equal to the number of trajectories simulated per state point. The more populated class is selected as the most representative of the state point. Noting that the classification results in two possible classes for each state point, and its reliability is further tested as follows. The class is regarded as a binary variable and the null hypothesis that the outcome of the classification results from the sampling of a binomial distribution with parameter 1/2 and with *N*_p_ trials is formulated. The null hypothesis is tested setting the *p* value to 0.01 and computing the probability of the classification outcome under the null hypothesis. The classification is regarded as reliable if this probability is found to be smaller or equal to *p*. In this way, each state point is classified as sinusoidal, constant or is not classified at all (*i.e.*, when the classification is regarded as not reliable).

## 4 Dynamics of a Janus particle near a wall

**Fig. 2:**
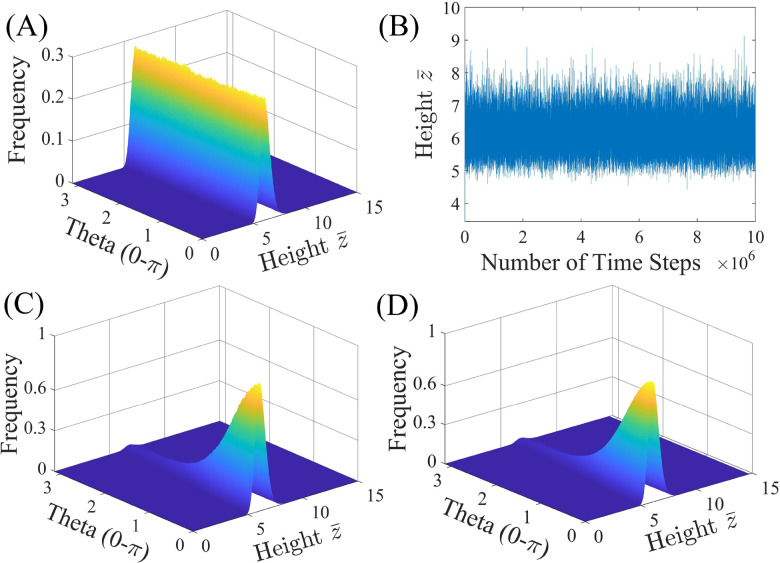
Impact of surface potential, *ψ*, on the probability density function (PDF) for a Janus particle of *R* = 1 μm without a cap (*δ* = 0), with surface potentials, *ψ*_1_ and *ψ*_2_, interacting with a wall of surface potential, *ψ*_w_ = −50 mV, using model 0: (A) PDF and (B) equilibrium height for *ψ*_1_ = *ψ*_2_ = −20 mV and model I: (C) PDF for *ψ*_1_ = −20 mV and *ψ*_2_ = −40 mV. (D) Boltzmann distribution prediction of the orientation distribution for the particle simulated in (C).

The behavior of a homogeneously charged (*i.e.*, constant surface potentials: *ψ*_1_ = *ψ*_2_) “Janus” particle of *R* = 1 μm without a cap (*δ* = 0) near a wall (SiO_2_) with negative surface potential of *ψ*_w_ = −50 mV is studied first to test the formulation and validity of the BD simulation, *i.e.*, model 0, and compared to a Janus particle without a cap (*δ* = 0), but with differing surface potentials (*ψ*_1_ ≠ *ψ*_2_), *i.e.*, model I, in Fig. 2.

Fig. 2(A) shows the expected PDF with equal probability for all orientations for a “Janus” particle with uniform surface potential (*ψ*_1_ = *ψ*_2_ = −20 mV, model 0) near a wall with *ψ*_w_ = −50 mV. The PDF is uniform because the driving force to rotate each face of the particle toward the wall is equal for both particle halves due to the identical surface potential. The particle rotates around its center, O, at an equilibrium height of *z̄* = 6.19 ± 0.45 as shown in the height trajectory of the particle in Fig. 2(B).

The PDF shown in Fig. 2(C) is obtained for a Janus particle where the surface potential of the two halves differ (model I) and are specified as *ψ*_1_ = −20 mV and *ψ*_2_ = −40 mV, while the wall surface potential is kept at *ψ*_w_ = −50 mV. Comparing the PDFs in Fig. 2(A) and (C), it is apparent that the particle stays at a similar equilibrium height but shows a bias in its configuration, *i.e.*, the particle tends to orient the face of lower surface potential, face 1, toward the wall (*θ* = 0) due to the greater surface potential gradient. Calculation of the equilibrium height reveals *z̄* = 6.32 ± 0.50 slightly increased from the particle with even surface potential. Careful inspection of the height distribution reveals that the particle is closer to the surface when face 2 is rotated away from the wall than when rotated toward the wall (*z̄*_max,0_ = 6.07 *vs. z̄*_max,π_ = 6.70) explaining the increased average equilibrium height value.

The dynamics of a Janus particle with the total potential interaction energy of *U*_tot_(*z*,*θ*) should follow the Boltzmann distribution prediction for which the probability distribution of various configurations is given as 
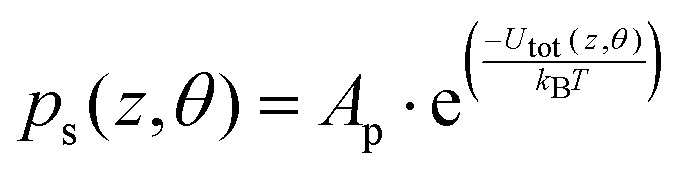
 with *A*_p_ chosen such that 
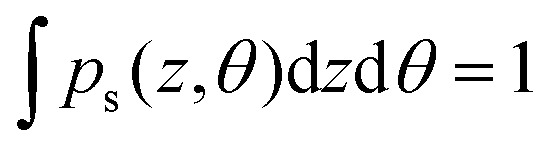
. Fig. 2(D) shows the Boltzmann distribution prediction for the same parameters used in the simulation of the PDF shown in Fig. 2(C). Comparison of Fig. 2(C) and (D) shows agreement thereby inferring the accuracy of the BD simulation methodology used here. Note data shown in the ESI† indicate that this holds true also for the plain particle model (see the ESI,† Section S3).

## 5 Dynamics of a bottom-heavy Janus particle near a wall

After evaluating the accuracy of models 0 and I for a uniformly charged “Janus” particle with equal surface potentials (*ψ*_1_ = *ψ*_2_) and the simplest Janus particle with differing surface potentials (*ψ*_1_ ≠ *ψ*_2_), respectively, model II introduces the effect of the cap, here made of platinum (*ρ*_Pt_ = 21.45 g cm^−3^), creating a bottom-heavy Janus particle. The weight of the cap and its geometry are incorporated in the simulations as described in Section 2. In this context, the cap of the particle (*i.e.*, its bottom-heaviness) contributes to the rotational dynamics of the particle and tends to rotate the cap toward the wall to antiparallel align the particle director (***n***_p_) with the wall's director (***n***_w_) *via* the corresponding torque of *N*_g_.

**Fig. 3:**
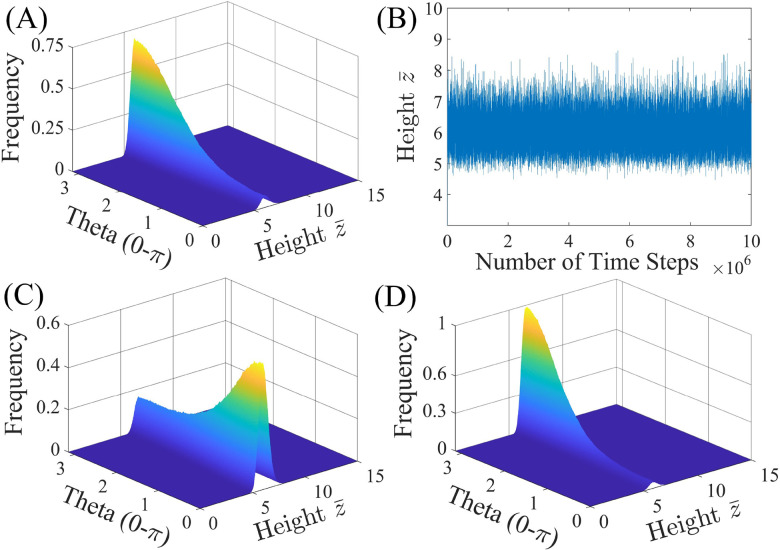
Impact of cap thickness and surface potential on the probability density function (PDF) of a bottom-heavy Pt-capped Janus particle of *R* = 1 μm with cap thickness, *δ*, and surface potentials, *ψ*_1_ and *ψ*_2_, interacting with a wall of surface potential, *ψ*_w_ = −50 mV using model II: (A) PDF and (B) equilibrium height *z̄* for *ψ*_1_ = *ψ*_2_ = −20 mV and *δ* = 10 nm. (C) Same as (A) with *ψ*_2_ = −40 mV. (D) Same as (C) with *δ* = 30 nm.

Fig. 3 shows the effect of incorporating a Pt cap (*δ* > 0) in the dynamical behavior of a particle with *ψ*_1_ = *ψ*_2_ and a Janus particle (*ψ*_1_ ≠ *ψ*_2_) with varying *δ*. Generally speaking, the bottom-heavy Janus particle stays at an equilibrium height and fluctuates with different preferred configurations near the wall due to an interplay of the effects of the cap and surface potentials. The PDF in Fig. 3(A) shows that for the case in which *ψ*_1_ = *ψ*_2_ (the wall potential is kept at *ψ*_w_ = −50 mV), the particle prefers rotating the face with the cap, face 2, toward the wall (*θ* = π). Furthermore, according to the height trajectory (Fig. 3(B)), the bottom-heavy Janus particle fluctuates at an equilibrium height of *z̄* = 6.09 ± 0.46 slightly smaller (and therefore closer to the wall) compared to the “Janus” particle with uniform surface potential in Fig. 2(B) due to the additional gravitational force of the cap.

Fig. 3(C) displays the PDF for the case in which the cap weight and the more negative surface potential on the same particle face, face 2, exert competing demands on the particle orientation; the cap forces face 2 towards the wall (*θ* = π) due to the gravitational torque, while the more negative surface potential forces face 2 away from the wall (*θ* = 0). For *δ* = 10 nm, the surface potential gradient dominates over the cap torque, and the particle exhibits a preference for rotation of face 2 away from the wall. The average height is found to be *z̄* = 6.37 ± 0.51, with the preferred height for the cap-up configuration (*θ* = 0) peaking at *z̄* = 5.99, while the particle in cap-down configuration (*θ* = π) is found most often at *z̄* = 6.70, in good agreement with the stronger repulsion between the wall and face 2. An increase in cap thickness to *δ* = 30 nm leads to the dominance of the gravitational cap torque that quenches the rotational motion of the particle to a mostly cap-down configuration (*θ* = π) as shown in Fig. 3(D). The average equilibrium height of the particle during the simulation is *z̄* = 6.50 ± 0.45 with cap-up and cap-down configuration heights peaking at *z̄* = 5.80 and 6.51, respectively. It is clear that the dynamic rotational behavior and the height of a bottom-heavy Janus particle depend strongly on the interplay of cap and surface properties. The complexity of this interaction is increased further by the introduction of shear flow and particle radius variations discussed in the next section revealing an intricate Janus particle flow behavior near a wall.

## 6 Dynamics of a bottom-heavy Janus particle near a wall under shear flow

In model III, the bottom-heavy Janus particle is subjected to shear flow with strain rate *γ̇* acting on the particle center, O. Initial simulations are performed with a strain rate of *γ̇* = 6 s^−1^ for sets of three Janus particles with *R* = 1 μm, *δ* = 86 nm, *ψ*_1_ = −20 mV, *ψ*_2_ = −40 mV, and *ψ*_w_ = −50 mV. Data for translation in *x̄*, *θ*, and *z̄* as a function of *t̄* are provided for the three particles in the ESI,† Section S4 (Fig. S7, top left). The three traces shown for *x̄*(*t̄*) indicate that the Janus particles move forward in the direction of the flow. The *θ*(*t̄*) traces show that each Janus particle starts in a unique, random orientation. Over time, *θ* values fluctuate between 0 and 2π in a cyclic fashion implying that the Janus particle rotates from a cap-down orientation (*θ* = 0) to a cap-up orientation (*θ* = π) back to a cap-down orientation (*θ* = 2π). Note that the particle orientation *θ* = 0 is equivalent to *θ* = 2π resulting in the abrupt change in *θ* at the end of each cycle. The *z̄*(*t̄*) traces closely follow the *θ*(*t̄*) traces with the particles at a high *z̄* when the particles are cap-down and a lower *z̄* when the particles are cap-up. A change in particle radius to *R* = 4 μm at the same *γ̇* = 6 s^−1^ and cap thickness, and for the same simulation length shows the expected longer distance *x̄*(*t̄*) and overall smaller *z̄* values with the oscillations in *z̄* more clearly matching the particle rotation (ESI,† Fig. S7, bottom left). Decrease of *γ̇* to 1 s^−1^ (ESI,† Fig. S7, top and bottom right) leads to a different *θ* behavior for both Janus particle sizes, where the Janus particle rotates until it assumes a constant *θ* value, *i.e.*, the strain rate is not strong enough to induce rotation (*ω* < *ω*_0_) and the particle slides along the wall with constant *θ*. The distinct behavior observed in the cap rotation, *i.e.*, *θ*(*t*), between the two particle sizes and as a function of *γ̇* prompts a careful search of the *γ̇*,*δ*-parameter space for *R* = 0.75, 1, 2, and 4 μm with *ψ*_1_/*ψ*_2_/*ψ*_w_ combinations of −20/−20/−20 mV, −20/−20/−50 mV, −40/−20/−20 mV, −40/−20/−20 mV, −20/−40/−20 mV, and −20/−40/−50 mV (see the ESI,† Section S5 and Fig. S8–S12). The findings from this parameter scan are summarized here.

**Fig. 4:**
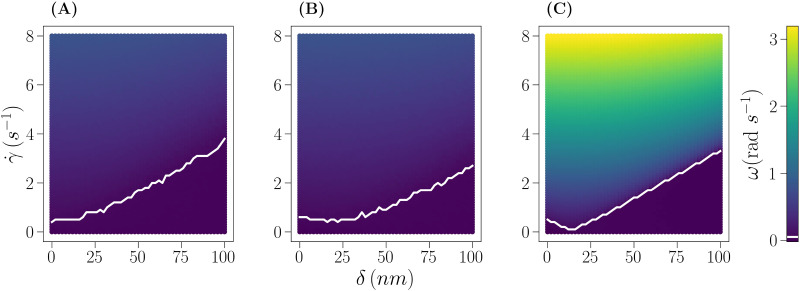
Angular velocity maps for a bottom-heavy Janus particle under varying shear flow (*γ̇* = 0–8 s^−1^) and cap thickness (*δ* = 0–100 nm) using model III for: (A) *R* = 1 μm, *ψ*_1_ = −20 mV, *ψ*_2_ = −20 mV and *ψ*_w_ = −50 mV. (B) As in (A), but *ψ*_2_ = −40 mV. (C) As in (B), but *R* = 4 μm. The color represents the average angular velocity as displayed by the color bar on the right. The transition line (white) is drawn at *ω* = 0.05 s^−1^.

Fig. 4 displays the angular velocity *ω* (see Section 3) of a Janus particle with the same *ψ*_1_, *ψ*_2_ and *ψ*_w_ values considered in Section 5 (Fig. 3) for the same particle radius *R* = 1 μm (Fig. 4(A) and (B)) and for a larger particle with radius *R* = 4 μm and surface potentials *ψ*_1_ = −20 mV, *ψ*_2_ = −40 mV and *ψ*_w_ = −50 mV (Fig. 4(C)) for cap thicknesses *δ* = 0–100 nm and strain rates *γ̇* = 0–8 s^−1^. All systems display the transition between a rotating (*ω* > *ω*_0_) and a non-rotating behavior (ω<ω_0_) described above as marked by the white line in Fig. 4. Above the transition line, *ω* increases with increasing *γ̇* at a constant cap thickness *δ*, whereas at constant *γ̇* and increasing *δ*, the change in *ω* is non-trivial. As the distance between the particle center, O, and the top of the cap, *i.e.*, the lever arm, grows linearly with *R* at a given *δ*, the torque acts more effectively on large particles, leading to the higher values of *ω* observed at the larger *R* (see colors in Fig. 4(A), (B)*vs.*Fig. 4(C)).

The onset of a non-negligible angular velocity at *δ* = 0 follows the expected trends by increasing from Fig. 4(A)–(B) due to the higher surface potential asymmetry and decreasing from Fig. 4(B) and (C) due to the increase in particle radius. However, once the cap is introduced (*δ* > 0) and increased in thickness, the transition line shows a distinct behavior for each system, ranging from a monotonic increase (Fig. 4(A)) to a slight decrease followed by a slow increase (Fig. 4(B)) and sharp decrease followed by a linear increase. The transition line, a locus of points in the diagram of the form (*δ*,*γ̇*), fully characterizes the transition: above (below) the locus the system is in the rotating (non-rotating) state. For example, if two points of the locus (*δ*_1_,*γ̇*_1_) and (*δ*_2_,*γ̇*_2_) are chosen such that the system is at the transition at those points, it is true for (almost) all systems that if *δ*_1_ < *δ*_2_, then *γ̇*_1_ < *γ̇*_2_. In other words, it is (almost) always true that increasing the cap thickness requires a stronger shear flow for the rotating phase to be entered (see Fig. 4(A) and the ESI,† Section S5). The only exception to this behavior is observed for Janus particles whose heavy side (face 2) is more repelled (*ψ*_2_ = −40 mV) by the wall then the light side (face 1, *ψ*_1_ = −20 mV) as shown in Fig. 4(B) and (C) (see also the ESI,† Section S5, Fig. S8 and S9). In this case, it is observed that *δ*_1_ < *δ*_2_ results in *γ̇*_1_ ≥ *γ̇*_2_ for sufficiently small *δ* (lower left corner). This behavior is persistent across different particle sizes and is more discernible for particles with *R* ≥ 2. A different way of describing this phenomenon is to see the locus of points as a set of values of *γ̇* that is a function of δ. In this perspective, it can be stated that *γ̇* is (almost) always a growing function of *δ*, with an exception at small *δ* when the surface potential asymmetry (*ψ*_1_ less negative than *ψ*_2_) makes the heavy face, face 2, more repelled by the wall. A minimum in the transition line then represents the cap thickness at which the opposing cap torque and surface potential gradient balance each other.

Surprisingly, the location of the non-rotating region depends only weakly on the particle radius, surface potential balance, and cap thickness. This behavior is explained by taking into account the complex interplay between particle radius, surface potential, and cap thickness, which together determine – for a given strain rate – the average distance *z̄* between the particle and the wall (see the ESI,† Section S5, and Fig. S10). For example, the strain rate is less effective when a more negative *ψ*_2_ than *ψ*_1_ results in a low *z̄*, but is more effective when a large *R* and/or a strong asymmetry in the particle's mass distribution due to a large *δ* also result in a low *z̄*.

At fixed *R* (Fig. 4(A)*vs.*Fig. 4(B)), an increase in electrostatic repulsion only slightly shifts the transition line to lower *γ̇* values, as it pushes the bottom-heavy particles farther from the wall, thus favoring their rotation. On increasing *R* (Fig. 4(B)*vs.*Fig. 4(C)), the lever arm grows, favoring the rotating behavior at a given *γ̇*, but this kind of increase is compensated by an increase in particle weight, which brings the particle closer to the wall, thus disfavoring the particle rotation due to the electrostatic repulsion. The previous argument helps to clarify the aforementioned complex dependence of *ω* on *δ* at constant *γ̇*. In particular, a non-monotonic behavior is observed when the repulsion strength between the wall and the heavy side of the particle is strong. This is because, as a consequence of the electrostatic repulsion, the particle is further away from the wall when the heavy side is closer to it. This effect interplays with the previously noted ones and triggers the rotation of particles with thin caps (including the *δ* = 0 case) at values of *γ̇* that would not be sufficient to trigger it in the absence of the surface potential asymmetry: the surface potential asymmetry pushes the particle upward enough to allow *γ̇* to act more effectively on it, hence triggering the rotational behavior.

**Fig. 5:**
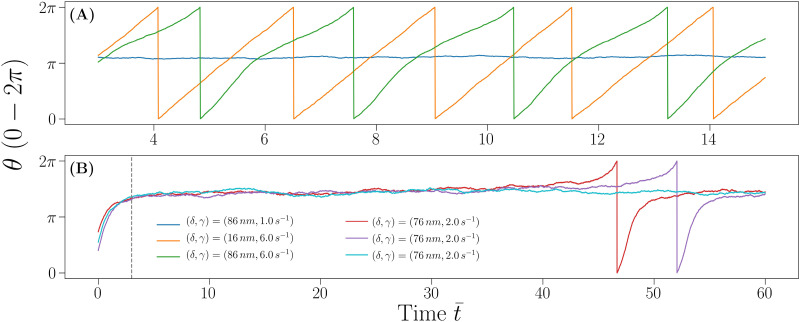
Representative angular trajectories for a system with *R* = 4 μm, *ψ*_1_ = −20 mV, *ψ*_2_ = −40 mV, and *ψ*_w_ = −50 mV. (A) Angular trajectories in a short-time window for state points specified in the legend shown in (B). (B) Angular trajectories in a long-time window for systems whose parameters are given in the legend. Note that the three trajectories shown in (B) correspond to different simulations of the same system. The dashed vertical line is the equilibration time after which the data are analyzed.

The phenomenology is, however, richer than what can be deduced from Fig. 4 only. A visual inspection of individual trajectories (Fig. 5) for the same system under different conditions shows evident differences. All curves are for *R* = 4 μm, *ψ*_1_ = −20 mV, *ψ*_2_ = −40 mV and *ψ*_w_ = −50 mV, *i.e.*, they correspond to state points in the angular velocity map shown in Fig. 4(C). In Fig. 5(A), the curve for *δ* = 86 nm and *γ̇* = 1.0 s^−1^ (blue) is an example of a trajectory sampled in the non-rotating phase and indeed the particle orientation simply experiences Brownian fluctuations. On increasing the shear rate to 6.0 s^−1^ while reducing the cap thickness to 16 nm (orange), the particle enters the rotating phase. The trajectory shows a growth of *θ* with time that is well approximated by a linear function, indicating a constant angular velocity. On increasing the cap thickness back to 86 nm (green), the behavior of *θ*(*t̄*) changes significantly: the first half of the rotation, for 0 ≤ *θ* < π is much faster than the second half. This change in angular velocity happens because the cap is heavy so gravity tends to keep it close to the wall and more work is required for the torque to fully rotate a particle in the opposite orientation, where the heavy side is aligned with the wall normal ***n***_w_ (*θ* = 0). This behavior is ascribed to an oscillating trend of the angular velocity, which is higher in the first part of the rotation and lower in the second. The observation motivates the classification introduced in Section 3. Fig. 5(B) illustrates a difficulty encountered by the classification. The curves in Fig. 5(B) show a mixed behavior despite corresponding to different simulations of the same state point in the angular velocity map. One of the curves (red) shows a single rotation over the whole temporal window of the numerical integration (*t̄* ≈ 47), the purple curve also shows a single rotation, but almost at the end of the integration (*t̄* ≈ 52) and the cyan curve does not show a rotation at all, despite the fact that a rotation would be observed if a longer numerical integration time were used. However, there is not a length of the numerical integration time that guarantees observation of at least one rotation for systems that exhibit rotations and not to observe rotation at all for systems that do not undergo rotations. When the model parameters are such that the rotational behavior exists, but the angular velocity is small, two different scenarios can be observed: very slow rotations with constant angular velocities, or the limit behavior of the sinusoidal angular velocity previously described.

Fig. 5(B) is an example of this kind of limit behavior: as shown by the red curve, the time it takes to complete the first half of the rotation is extremely short, due to the heavy cap driving face 2 toward the wall, while the time it takes to complete the second half of the rotation is exceedingly large as shear flow and surface potential repulsion must overcome the gravitational torque from the cap. This phenomenon can be described by the first passage time distribution as a parametric function of the orientation *θ*:^55^ this kind of function describes the probability that *θ* reaches a certain (parametric) value in a time t*, which is the random variable of the distribution. Stochastic processes near a phase transition are typically characterized by a fat tailed distribution of the first passage time^56^ and the results presented here suggest that the system under analysis makes no exception: for a large value of the parameter *θ*, the distribution would likely appear heavy tailed, indicating that no matter how long the numerical integration is, the probability to not observe a full rotation would still be non-zero.

Note the regime of constant angular velocity can also be seen as a purely rotational regime, where the translational motion of the particle is strongly correlated with its orientation, as one expects for a ball rotating on a plane, while the variable angular velocity, induced by surface potential imbalance and asymmetry of the mass distribution, can be seen as a type of motion in which the correlation between orientation and position along the plane is increasingly weaker as the linearity in *θ*(*t*) is lost, due to a “slipping” behavior in which the particles translate while rotating independently of the translating motion.

**Fig. 6:**
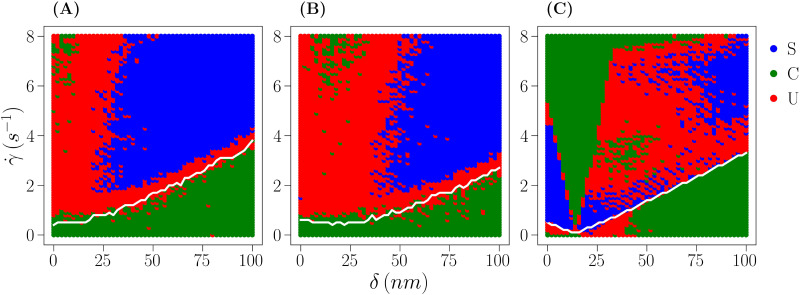
State diagram of a bottom-heavy Janus particle under varying shear flow (*γ̇* = 0–8 s^−1^) and cap thickness (*δ* = 0–100 nm) for: (A) *R* = 1 μm, *ψ*_1_ = −20 mV, *ψ*_2_ = −20 mV and *ψ*_w_ = −50 mV. (B) Same as in (A), but *ψ*_2_ = −40 mV. (C) Same as in (B), but *R* = 4 μm The white line is the same transition line drawn in Fig. 4, and colors represent the classification of each state point as described by the legend on the right. Symbols in the legend stand for sinusoidal (*S*), *i.e.*, non-constant angular velocity, constant (*C*), *i.e.*, constant angular velocity, and unclassified (*U*).

The results of the classification are shown as state diagrams in Fig. 6 and in the ESI,† Section S6. The transition line between the rotating and the non-rotating behavior is identified by the white transition line. Almost all the state points below it are ascribed to constant angular velocity (green state point) with a small region of unclassified states (red state point) inside the non-rotating phase only for large particle radius, small cap thickness, and small shear flow. Just above the transition line, a region of unclassified states points is systematically observed. This is due to the phenomenon exemplified in Fig. 5(B) and explained in the previous paragraph: near the transition, some realizations appear as constant (cyan curve) and some are clearly not well fitted by a constant function (red and purple curves), resulting in state points that are not classified at all. Further above the transition line, at large cap thickness and strong shear flow, the system is systematically characterized by a sinusoidal angular velocity (blue state point). On progressively reducing the cap thickness, the system enters first a region of unclassified state points in which the distinction between the two behaviors is not simple as the difference in slopes between the two regimes of rotation is extremely small, but not small enough for the state points to be systematically ascribed to a constant behavior. On further reducing the cap thickness, a region of constant angular velocity is entered. This region displays an interesting behavior as the model parameters change: on reducing *R*, it systematically shifts to the left (Fig. 6(C)*vs.*Fig. 6(B) and ESI,† Fig. S12), it is observed to shift to the right (Fig. 6(A)*vs.*Fig. 6(B) and ESI,† Fig. S11, S12) if the surface potential of the heavy side of the particle is increased (in absolute value, at fixed *R*) and it shifts upward if the other side is overcharged with respect to the wall (ESI,† Fig. S11). See the ESI,† Section S6 for a more detailed analysis of the results.

Despite being apparently intricate, this behavior is relatively simple to understand. Constant but sinusoidal angular velocity is observed when the shear rate is strong enough to induce rotational motion (above the transition line), but the surface potential and density imbalances are not so strong that one side is more prone to be closer to the wall than the other. Hence, at comparable repulsion between the two sides and the wall (*ψ*_1_ = *ψ*_2_), only the cap induces asymmetry and this can be compensated when the cap is small and the shear flow is large. Reducing *ψ*_2_ to more negative potentials means that the light side of the particle is more likely to stay close to the wall, which is balanced by the cap pushing the particle in the opposite orientation, hence increasing the width of the constant regime. When the light side is strongly repelled by the wall, the region shifts upward as a given asymmetry in the mass distribution requires a strong shear rate to compensate the repulsion at small cap sizes. Furthermore, when the constant behavior region is observed at large cap thickness, it is also seen that a new sinusoidal region exists on its left, when both cap thickness and shear rate are small (ESI,† Fig. S11 and S12, bottom row).

Summarizing the behavior of bottom-heavy Janus particles under shear flow, it can be stated that outside of the rotating region the system is characterized by a sinusoidal angular velocity if the cap is thick enough to induce a strong asymmetry. Reducing the cap thickness at a given shear rate implies reducing the variability in *ω*(*t̄*) up to completely balancing the speed of rotation in the two half periods and, possibly, on further reducing the cap thickness a sinusoidal angular velocity is observed again (especially for large particles), as the surface potential imbalance is not compensated by the weak flow. Data provided in the ESI† (Sections S5 and S6) shows that this behavior is persistent across different model settings.

Last but not least, in model IV, the impact of hydrodynamic friction is considered taking into account the Janus particle's closeness to the wall for the *R* = 1 μm and *R* = 4 μm systems discussed at the beginning of this section (*δ* = 86 nm, *ψ*_1_ = −20 mV, *ψ*_2_ = −40 mV, and *ψ*_w_ = −50 mV). Based on Goldman *et al*.'s report,^49^ frictional factors *f*_*x*_ and *f*_*θ*_, which are functions of *z̄*, are determined and introduced into eqn (5) and (7), respectively. For a bottom-heavy Janus particle of *R* = 1 μm and cap thickness of 86 nm at an average height of *z̄* = 6 ± 1, *f*_*x*_ = 0.9887 and *f*_*θ*_ = 0.9882 indicating that these particles experience very little hydrodynamic friction. The situation changes when the particle size is increased to *R* = 4 μm and the particles display an average *z̄* = 3 ± 0.5 resulting in an *f*_*x*_ = 0.8342 and *f*_*θ*_ = 0.8428. Inclusion of these friction factors in the simulation reduces the angular velocity of the *R* = 4 μm Janus particle resulting in approximately 20% fewer rotations as shown in the ESI,† Section S7, Fig. S13, while the *R* = 1 μm Janus particle is too far away from the surface to be substantially impacted by friction. Note that Goldman *et al.*'s^49^ friction coefficients were calculated for a uniform, neutrally buoyant sphere near a plane wall under simple shear flow. Geonzon *et al.*^57^ experimentally verified Goldman *et al.*'s^49^ friction coefficients for unmodified SiO_2_ spheres (*R* = 1.25 μm) under shear flow using optical tweezers. More recently, the translational and rotational drags of a Janus particle close to a wall without shear flow were determined using fluorescently labeled Pt-coated MF beads of *R* = 1.24 ± 0.05 μm and were also found to be in good agreement with Goldman *et al.*'s values.^58^ Experimental verification of the effect of shear flow on the hydrodynamic drag force for Janus particles near a wall would be desirable. Unfortunately, Janus particles with metal caps experience thermophoresis in optical traps due to the heating of the metal cap making such measurements non-trivial.^59^

## 7 Conclusion

The behavior of a bottom-heavy, charged Janus particle near a charged wall under shear flow is fully rationalized, providing insights into how both confinement and shear flow can be utilized to manipulate the particle's translational and rotational motion.

The dynamical behavior of a Janus particle is described using a model based on gravitational and electric double layer interactions. The simplest Janus particle model, model I, shows that the Janus particle without a cap of differing density tends to orient toward the wall with the face of lower surface potential because of the greater potential gradient. For the case of a uniformly charged bottom-heavy Janus particle, *i.e.*, a particle with a cap of mismatched density but uniform surface potential, model II, the cap quenches the rotational motion of the particle leading to a preference for a cap-down orientation adjacent to the wall in good agreement with existing literature.^25,26^ Adjustment of the caps surface potential leads to a competition between gravitational torque and electrostatic repulsion in which the position of the particle above the wall and its rotational behavior become a function of radius, surface potential, and cap thickness.

The relationship is further complicated by the introduction of shear flow at small Pe_p_ in model III. Thorough analysis of the particle behavior for a range of shear flows (0–8 s^−1^), cap thicknesses (0–100 nm), radii (0.75–4 μm), and surface potential combinations (*ψ*_w_ = −20, −50 mV and *ψ*_1,2_ = −20, −40 mV) reveals complex angular velocity profiles for the bottom-heavy Janus particle with regions of no rotation, constant rotation, and time-varying rotation. Use of statistical analysis leads to state diagrams that reveal *δ*,*γ̇*-combinations for which the passive, bottom-heavy Janus particle shows constant angular velocity with and without rotation and a sinusoidal angular velocity. The latter state can be described as the particle reaching a cap-down orientation followed by a sliding event before completing its rotation where the sliding time can be engineered and therefore is of potential interest when patch–wall interactions need to be timed in applications such as drug delivery or environmental remediation. Finally, we note that hydrodynamic friction, model IV, effectively reduces the particle's ability to rotate by ≈20% when the particle is close to the wall, which occurs for large radii and/or thick caps at high shear rates, but can be neglected for small particles and/or thin caps.

For future work, it is worth noting that the choice of orientation-dependent factors 
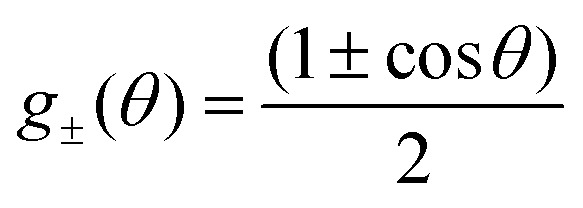
 balances simplicity and numerical efficiency in the BD model employed here. A more rigorous formulation could be developed based on the Janus sphere–sphere potentials presented by Popov and Hernandez.^60^ A detailed discussion on the selection of weighting factors and their influence on the torque is provided in Section S8 of the ESI.†

## Author contributions

Z. J., U. C., and I. K. discussed and conceptualized the project. Z. J. formulated the theoretical model, developed the initial code and provided a first draft of the manuscript as part of her PhD thesis.^61^ I. K. revised the theoretical model and finalized the code. Numerical simulations were performed by Z. J. I. K. and D. N. D. N. conceptualized, implemented, and performed the statistical analysis needed for the angular velocity maps and state diagrams. D. N., E. B., and I. K. discussed the interpretation of angular velocity maps and state diagrams and revised the manuscript during I. K.'s sabbatical stay in E. B.'s group. All authors have read and approved the final version.

## Conflicts of interest

There are no conflicts to declare.

## Supplementary Material

SM-021-D5SM00229J-s001

## Data Availability

Data supporting the statements and conclusions made in the manuscript are included as part of the ESI.† Additional data is available upon reasonable request from the corresponding authors.
