# Supplementary material for: Dynamics of a bottom-heavy Janus particle near a wall under shear flow
Source: Soft Matter. 2025 Jun 18;21(28):5773–84. doi: 10.1039/d5sm00229j (PMC12198726; doi:10.1039/d5sm00229j)
Supplement: SM-021-D5SM00229J-s001 [file SM-021-D5SM00229J-s001.pdf]

Supplementary Information (SI) for Soft Matter.
This journal is © The Royal Society of Chemistry 2025

Supporting Information: Dynamics of a Bottom-Heavy Janus Particle Near a Wall Under Shear Flow

Zohreh Jalilvand,[a] Daniele Notarmuzi,*[b] Ubaldo M. Córdova-Figueroa,[c] Emanuela Bianchi,[b,d] and Ilona Kretzschmar*[a]

The supporting information document provides additional details on the model for a plain, charged particle near a charged wall (Section S1), simulation details for data presented in the manuscript (Section S2), validation of the model introduced in Section S1 (Section S3), impact of shear flow on particle behavior (Section S4), angular velocity maps (Section S5), state diagrams (Section S6), data illustrating the impact of hydrodynamics (Section S7), and illustration of the impact of EDL torque (Section S8).

S1 BD Simulation Details for a Single Charged Particle Near a Charged Wall

A spherical particle of radius R is considered. The particle has a uniform surface charge ψ_p, is suspended in a density-mismatched Newtonian fluid with viscosity μ, an electrostatic permittivity ε_0, and an inverse Debye screening length κ that is determined by the ionic strength I of the fluid. The thermal energy of the system is represented by k_BT, where k_B is Boltzmann's constant. Importantly, the particle is bounded by a charged wall (ψ_w). Assuming a constant charge condition and using height z to describe the separation distance of the particle surface from the wall, the surface interaction energy per unit area, W, resulting from the electric double layer interaction, is expressed by equation (S1):[1]

$$W = \varepsilon\kappa \frac{2\psi_p\psi_w e^{-\kappa z} + (\psi_p^2 + \psi_w^2)e^{-2\kappa z}}{1 - e^{-2\kappa z}} \,. \tag{S1}$$

Equation (S1) is based on the Poisson-Boltzmann theory. The force, F, between two interacting objects, can be related to the surface interaction energy, W, via the Derjaguin approximation, equation (S2):

$$F = 2\pi R_{eff} W \,, \tag{S2}$$

where the effective radius, R_{eff}, for the case of a sphere interacting with a planar substrate is equal to the particle radius R. Integrating equation (S2) over the height z, provides the electrostatic double-layer interaction potential, U_{EDL}, as shown in equation (S3).

$$U_{EDL} = 2\pi R\varepsilon \left[\psi_p\psi_w \ln\frac{1 + e^{-\kappa z}}{1 - e^{-\kappa z}} - (\psi_p^2 + \psi_w^2)\frac{\ln(1 - e^{-2\kappa z})}{2}\right] \tag{S3}$$

[a] Address: Department of Chemical Engineering, City College of New York (CCNY), City University of New York (CUNY), 140th Street & Convent Avenue, New York, New York 10031, United States; E-mail: kretzschmar@ccny.cuny.edu
[b] Address: Institute for Theoretical Physics, Technische Universität Wien, Wiedner Hauptstraße 8-10, A-1040, Vienna, Austria. E-mail: daniele.notarmuzi@tuwien.ac.at
[c] Address: Department of Chemical Engineering, University of Puerto Rico-Mayagüez, Mayagüez, PR 00681, USA.
[d] Address: CNR-ISC, Uos Sapienza, Piazzale A. Moro 2, 00185, Roma, Italy

A theoretical model is employed on the basis of the stochastic Langevin equation to probe the dynamics of the particle interacting with the wall according to equation (S4).

$$\frac{dr_i}{dt} = u^\infty(r_i) + \frac{1}{\gamma}(F_i^B + F_i^{nh}) \tag{S4}$$

Here, $u^\infty(r_i)$ is the velocity of the ambient fluid at particle position $\{r_i\}$ and γ is the drag coefficient for a perfect sphere, $6\pi\mu R$. The non-hydrodynamic force, F_i^{nh}, includes any surface forces or external body forces, i.e., inter-particle interaction forces, that are a function of $\{r_i\}$. F_i^B is a random fluctuating force acting on the particle that describes Brownian diffusion and renders equation (S4) a stochastic differential equation. F_i^B is assumed to represent a Gaussian stochastic process with the following moments:

$$\{F_i^B\} = 0\,, \tag{S5}$$

$$\langle\{F_i^B(t)\}\{F_i^B(t')\}\rangle = 2\gamma k_B T\delta(t-t')\,, \tag{S6}$$

where T is the absolute temperature and $\delta(t-t')$ is the Dirac delta function. The magnitude of F_i^B is a function of T and the drag force, which follows the fluctuation-dissipation theorem. For a colloidal particle, F_i^B mimics the impact of the ambient fluid's fluctuating molecules on the particle and is expressed by the white noise function $\eta(t)$, which is a Gaussian stochastic process with moments $\langle\eta(t)\rangle = 0$ and $\langle\eta(t)\eta(t')\rangle = \delta(t-t')$. Note the standard white noise assumption for the Brownian force applies for the system studied here as the ratio of $\dot{\gamma}$ over the bath frequency ($\approx 10^{12}\,s^{-1}$) is less than 10^{-11}. For larger ratios, i.e., very large $\dot{\gamma}$ or more viscous fluids, a non-white noise more accurately describes the random fluctuations.[2] Additionally, the ratio of the advection to Brownian forces is expressed by the Péclet number based on the colloidal Brownian/diffusion relaxation, $Pe_p = \dot{\gamma}R^2/D_0$, where D_0 is the translational diffusivity of the isolated Brownian particle in bulk. Depending on the particle size and strain rate, Pe_p numbers vary over the range of $0 \leqslant Pe_p \leqslant 0.5 \times 10^4$. The sedimentation force and particle-wall interactions are incorporated into the model via equation (S4), within the framework of the Derjaguin-Landau-Verwey-Overbeek (DLVO) theory as non-hydrodynamic forces acting on the particle, F^{nh}, to describe the dynamical behavior of the particle.

S2 Details of numerical simulations and of statistical testing

Results presented in Figures 2 and 3 in the manuscript are obtained from Matlab simulations of $N_p = 30$ particles over $\bar{t} = 1 \times 10^4$ and $dt = 0.001$ resulting in 1×10^7 integration steps. Owing to the large number of integration steps and the short equilibration time (ca. 500 steps) needed for the particle, all orientations and heights are included in the averaging. The initial condition is randomized using Matlab's default seed by assigning to z a random value from a normal distribution with mean 3 and variance 1, while the initial condition on the particle orientation θ is drawn uniformly in the interval $[0, \pi]$. z and θ axes are divided into 800 and 600 bins, respectively.

Results presented in Section 6 of the manuscript are obtained by simulating $N_p = 50$ independent particles, each integrated from $t_0 = 0$ to $t_1 = 60$ and using $dt = 0.006$,

resulting in 1×10^4 integration steps. The equilibration time is set to $t_{eq} = 3$, which corresponds to removal of the first 500 integration steps from the data analyzed. The initial condition is randomized by assigning to x and y a random value from a normal distribution with mean 3 and variance 1, the initial condition on the particle orientation θ is drawn uniformly in the interval $[0, \pi]$. Note, however, that the system quickly reaches equilibrium so the dynamics observed at $\bar{t} \geq t_{eq}$ is completely independent from the initial condition.

Angular velocity maps (see Section S5) are obtained by computing the integral in equation (10) of the manuscript and averaging over the 50 particles simulated. For state diagrams (see Section S6), the period of the rotation of each particle is computed as $T = 2\pi/\langle\omega\rangle_t$. Subsequently, the number of bins is set to $\lfloor 12(t_1 - t_{eq})/T \rfloor$, corresponding to 12 bins per period, along the entire integration time. For particles with very small $\langle\omega\rangle_t$, i.e., very large periods, this results in a very small number of bins. If the number of bins is smaller than 100, it is manually set to 100. Note that the results of the classification might slightly depend on the assignment of the number of binds, especially in regions where the two behaviors are close. The resulting trajectories of the orientation averaged over time are used to compute the derivative to obtain the time-dependent angular velocity $\omega(\bar{t})$.

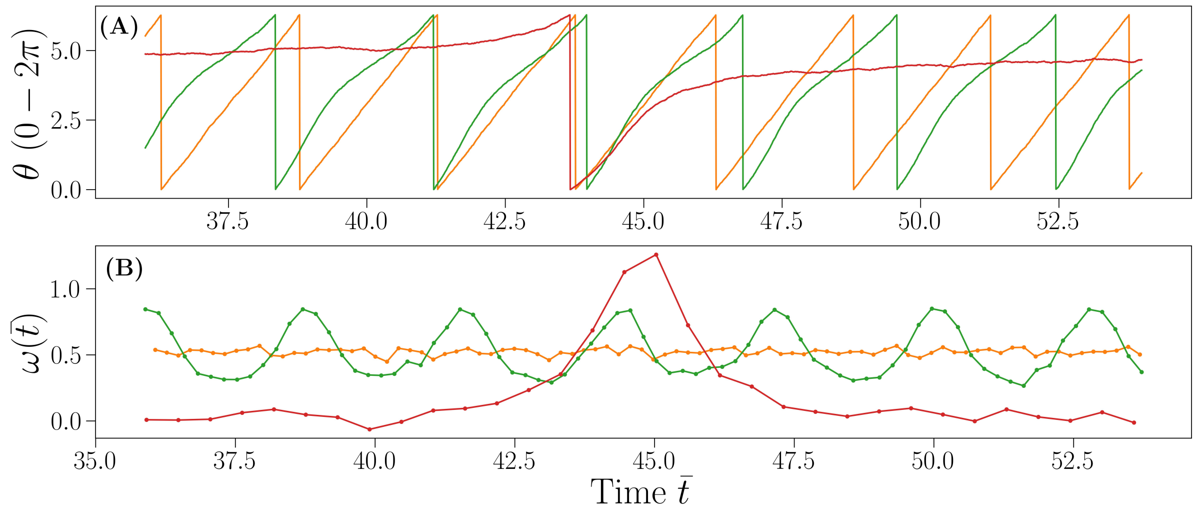

Figure S1: Representation of angular trajectories and corresponding time-dependent angular velocity. Top: angular trajectories for three states (orange and green from Figure 5A and red from Figure 5B) shown in Figure 5 of the manuscript. All curves are for $R = 4\,\mu m$, the orange curve is for $\delta = 16\,nm$ and $\dot{\gamma} = 6.0\,s^{-1}$, the green curve is for $\delta = 16\,nm$ and $\dot{\gamma} = 2.0\,s^{-1}$ and the red curve is for $\delta = 76\,nm$ and $\dot{\gamma} = 2.0\,s^{-1}$. Bottom: Time dependent angular velocity obtained from the three trajectories in top panel using the same color scheme.

Figure S1 shows three examples of $\omega(\bar{t})$. Note that the curves in the top panel are the same data shown in Figure 5 of the manuscript with matching colors (see caption). For each state point, all $\omega(\bar{t})$ are fitted to a constant and a sinusoidal model and the fit is compared using the Akaike Information Criterion,[3] i.e.,

$$AIK = 2k + n\log(ssr/n)\,, \tag{S7}$$

where k is the number of parameters in each model (one for constant, four for sinusoidal), n is the number of data points (which in the present context correspond to the number of distinct values of $\omega(\bar{t})$, i.e., the number of bins) and ssr is the sum of the squared difference between the data and the model (known as sum of squared residuals, hence ssr). The model with the lowest AIK is considered to be the best model for a trajectory. Referring to Figure S1, it is clear that the orange curve is best fitted by a constant model and, analogously, that the green curve is best fitted by a sinusoidal model. Example results for fits to the red curve are shown in Figure S2. Clearly, both the constant and

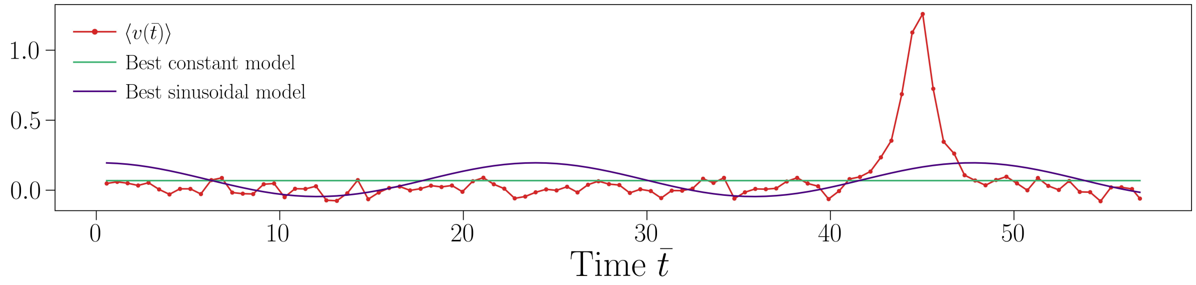

Figure S2: Fit to a trajectory at the boundary between rotating and non-rotating behavior. The red curve is the same data as the red curve in Figure S1, i.e., $R = 4\,\mu m$, $\delta = 76\,nm$ and $\dot{\gamma} = 2.0\,s^{-1}$. The light green curve is the best constant model that describes the data and the purple curve is the best sinusoidal model that describes the data.

sinusoidal models are bad fits. Nevertheless, the sinusoidal model turns out to be better because the peak associated with the only observed rotation introduces a very strong penalty for the constant model, which not only does not capture the peak well, but is also shifted upward by it and hence does not capture the constant part either. The sinusoidal model, instead, is able to better "absorb" the variations in the data and therefore turns out to be slightly better. Other realizations of the same state point, however, do not show the rotation and hence do not have a peak in $\omega(\bar{t})$, like the cyan trajectory shown in Figure 5B of the manuscript. Those trajectories are better fitted by the constant model. As this protocol is applied to each of the 50 particles simulated, it results in a binary classification of the form shown in Figure S3.

Figure S3 shows the number of particles classified as constant or sinusoidal for the three examples of Figure S1. A two-sided binomial test is applied to decide whether or not the classification is reliable. To this aim, a null hypothesis is made that the data come from the random sampling of a binary variable, i.e., that they come from $B(k, 50, 0.5)$, the binomial distribution with 50 trials (the number of particles simulated per state point) and success probability 0.5. The threshold of the p value is fixed at 0.01 and the confidence interval of the null hypothesis is computed. If the frequency of both classes falls inside such an interval, the null hypothesis cannot be rejected, the state point is not classified and is marked as a red state point. In the case where the null hypothesis can be rejected and the test result is assumed to be reliable, the state point is assigned to the more populated class (constant or sinusoidal).

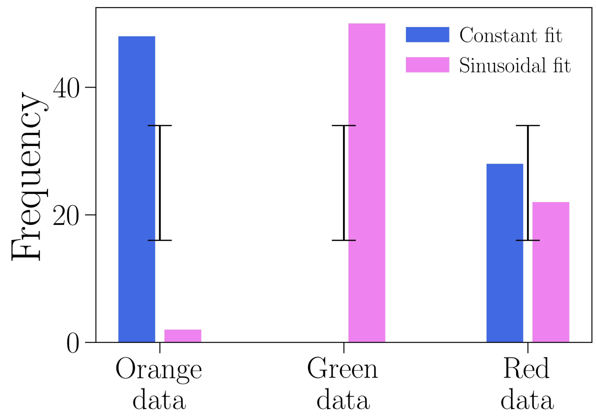

Figure S3: Outcome of classification of the state points examplified by the trajectories in Figure S1. The blue (pink) bar represents the number of particles classified as constant (sinusoidal). Black error bars represent the confidence interval of the null hypothesis.

S3 Dynamical behavior of the charged particle near a charged wall

It is essential to verify the accuracy of the governing equations introduced in Section S1 for the system of interest, i.e., for a negatively charged particle interacting with a negatively charged wall. Therefore, the modeled electrostatic double-layer interaction potential, U_{EDL}, derived in Section S1 is compared to published Total Internal Reflection Microscopy (TIRM) data. The TIRM experimental measurement shown in Figure 5A of Volpe et al.[4] was obtained using a polystyrene particle with radius $R = 1.45\,\mu m$ near a glass surface, density of $\rho_p = 1.053\,g/cm^3$, suspended in $300\,\mu M$ aqueous NaCl background electrolyte, and a Debye screening length $\kappa^{-1} = 17\,nm$ for the 1:1 electrolyte. Using the same parameters in equation (S3), the potential-distance relationship in Figure S4 is obtained and shows good agreement with Volpe et al.'s[4] TIRM data.

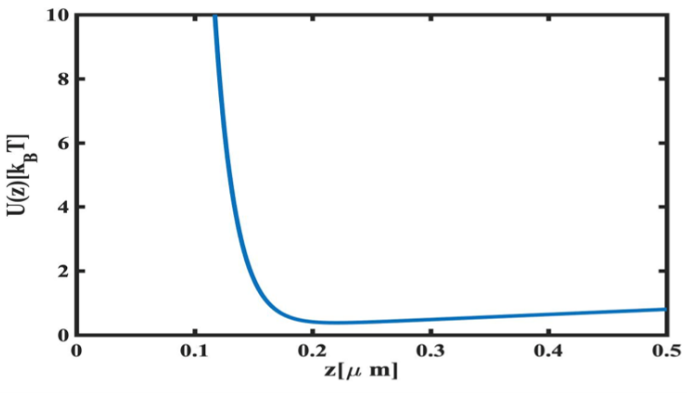

Figure S4: Interaction potentials for a negatively-charged polystyrene particle bounded by a negatively charged glass wall from equation (S3).

For a spherical particle with a total energy of $U_{tot}(z) = U_{EDL} + U_{gravity}$ in thermal equilibrium with the surrounding fluid that obeys the one-dimensional stochastic differential equation (S4), the probability distribution function (PDF) of the height of the particle is given by the Boltzmann distribution:

$$\rho_s(z) = A_p \cdot e^{[-\frac{U_{tot}(z)}{k_B T}]} \ , \tag{S8}$$

with A_p chosen such that $\int p_s(z)dz$=1. Hence, in another check of the validity of the technique and the methodology, Figure S5 shows the numerically calculated PDF (blue bars) of a SiO_2 particle with R $= 1\,\mu m$ suspended in DI water (Millipore, resistivity 18.2 MΩcm and viscosity $\mu = 1e^{-3} Pa\, s$ at 25 °C, $I = 1e^{-6}\, M$) at an equilibrium state overlaid with the theoretical Boltzmann distribution (red curve).

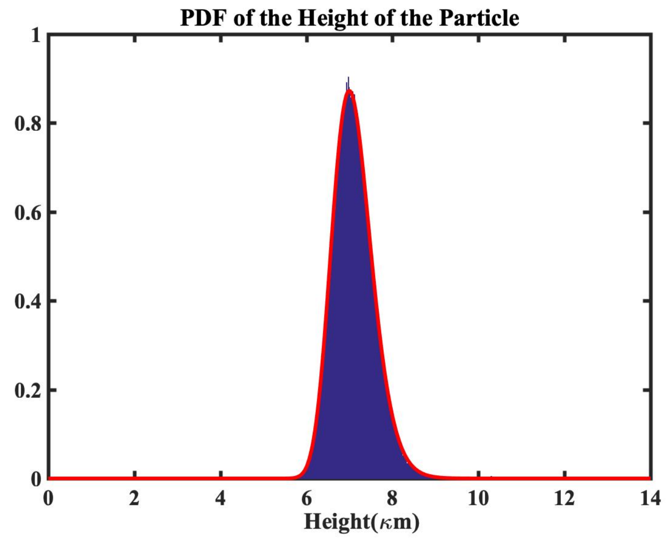

Figure S5: Comparison of the simulated PDF of the height of a SiO_2 particle of R $= 1\,\mu m$ above a negatively charged wall (blue histogram) and the theoretically prediction from Boltzmann distribution (red curve).

In order to describe the trajectory of a uniformly charged particle of radius R suspended in a fluid and bounded by a wall, the electrostatic double later interaction F_{EDL}, equation (S9), as well as the effective gravitational force F_g, equation (S10), are considered as non-hydrodynamic forces in equation (S4), (i.e., $F_i^{nh} = F_{EDL} + F_g^*$).

$$F_{EDL} = 2\pi R \varepsilon_0 \kappa \frac{2\psi_p \psi_w e^{-\kappa z} + (\psi_p^2 + \psi_w^2) e^{-2\kappa z}}{1 - e^{-2\kappa z}} \ , \tag{S9}$$

$$F_g = -m^*\, g \tag{S10}$$

where m^* is the effective mass of the particle, i.e., the density mismatch between the particle and the surrounding fluid is taken into account. Substituting equations (S9) and (S10) into equation (S4) for the F_i^{nh} term in the z direction and the Brownian force, F_i^B, with the Gaussian stochastic process, $\eta_z(t)$, discussed in Section S1, the final Langevin equation is obtained:

$$\gamma \frac{dz}{dt} = F_{EDL}(z, \psi_p, \psi_w) + F_g + \ell \eta_z(t) \quad \text{(S11)}$$

Equation (S11) is rendered dimensionless by measuring the length scale in units of Debye screening length κ^{-1}, i.e., $\bar{z} = \kappa z$, and the time scale in units of $T_0 = \frac{3\pi\mu R}{k_b T \kappa^2}$, i.e., $\bar{t} = t/T_0$.

Numeric integration of equation (S11) utilizing the Euler time integration scheme yields the trajectories of the Brownian particle bounded by the wall. Figure S6 displays the 1D height trajectory obtained for an SiO_2 particle of radius $R = 1\,\mu m$ bound by a negatively charged wall.

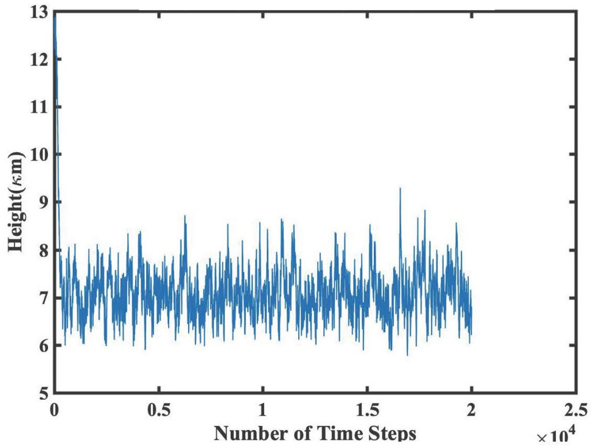

Figure S6: 1D trajectory of a SiO_2 particle near a wall fluctuating around the equilibrium height that matches with the probability distribution function shown in Figure S5.

Agreement of the model with published experimental TIRM data (Fig. S4) and overlap of the simulated height distribution with theoretically predicted Boltzmann distribution (Fig. S5) instills confidence that the BD simulations can successfully simulate the dynamics of a bottom-heavy Janus particle near a wall under more complicated conditions.

S4 Impact of Shear Flow and Radius on Particle Behavior in Model III

The behavior of a particle using Model III is summarized in Figure S7 for four specific state points. Surface charges are set to $\psi_1 = -20\,mV$, $\psi_2 = -40\,mV$ and $\psi_w = -50\,mV$, and $\delta = 86\,nm$. Each state point is represented by three panels (from top to bottom): non-dimensionalized particle distance from origin along x-axis $\bar{x}$, particle orientation θ and non-dimensionalized particle height $\bar{z}$ shown as a function of non-dimensionalized time $\bar{t}$. Figure S7 (top left quadrant) shows the state point for $R = 1\,\mu m$ and $\dot{\gamma} = 6.0\,s^{-1}$. Figure S7 (top right quadrant) shows the state point for the same radius, $R = 1\,\mu m$, but

a decreased shear flow, $\dot{\gamma} = 1.0\,s^{-1}$. Figure S7 (bottom left quadrant) shows the state point for $R = 4\,\mu m$ and $\dot{\gamma} = 6.0\,s^{-1}$, while Figure S7 (bottom right quadrant) shows the state point for the same radius, $R = 4\,\mu m$, but decreased shear flow, $\dot{\gamma} = 1.0\,s^{-1}$.

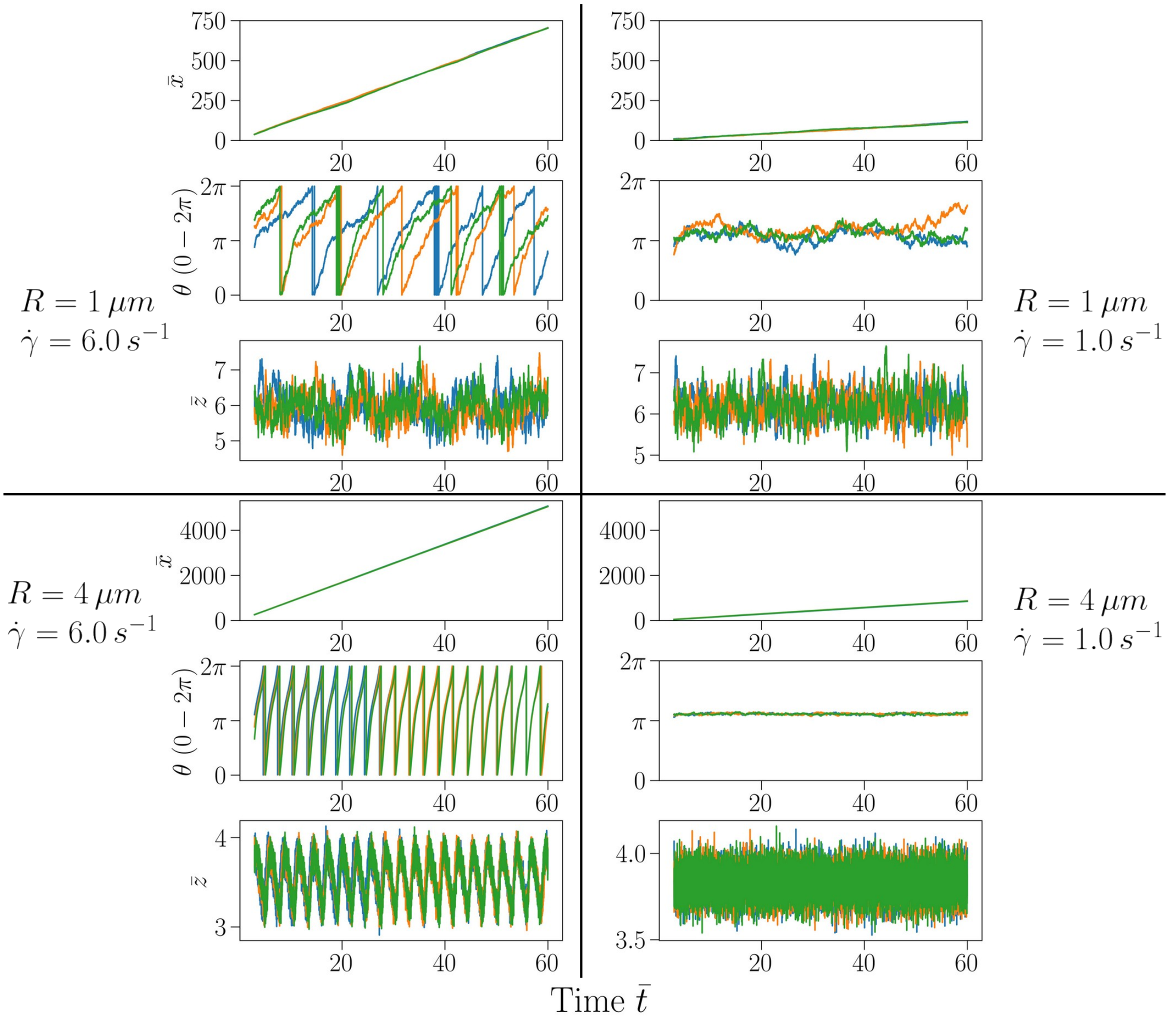

Figure S7: Overall behavior of a particle in Model III. Surface charges are set to $\psi_1 = -20\,mV$, $\psi_2 = -40\,mV$ and $\psi_w = -50\,mV$, and $\delta = 86\,nm$ for (top left quadrant) $R = 1\,\mu m$ and $\dot{\gamma} = 6.0\,s^{-1}$, (top right quadrant) $R = 1\,\mu m$ and $\dot{\gamma} = 1.0\,s^{-1}$, (bottom left quadrant) $R = 4\,\mu m$ and $\dot{\gamma} = 6.0\,s^{-1}$, and (bottom right quadrant) $R = 4\,\mu m$ and $\dot{\gamma} = 1.0\,s^{-1}$. Three panels for each state from top to bottom are: non-dimensionalized particle distance from origin along x-axis $\bar{x}$, particle orientation θ and non-dimensionalized particle height $\bar{z}$ shown as a function of non-dimensionalized time $\bar{t}$.

S5 Angular Velocity Maps and Particle Height for Model III

Angular velocity maps, ω-maps, are presented in this section as a function of δ and $\dot{\gamma}$ for a large class of systems. Specifically, three combinations of surface charge of the particle are considered, i.e., $(\psi_1, \psi_2) = (-20, -20)$, $(-20, -40)$, $(-40, -20)$, all expressed

in mV. For each of the combinations, two values of the wall charge $\psi_w = -20\,mV$ and $\psi_w = -50\,mV$ are considered, hence specifying six classes of systems as they emerge from the electrostatic properties of the particle and the wall. In each of the six classes, four particle radii $R = 0.75, 1.00, 2.00$, and $4.00\,\mu m$ are considered. The resulting ω-maps are shown in Figures S8 and S9 for $\psi_w = -20\,mV$ and $\psi_w = -50\,mV$, respectively, with R increasing from left to right.

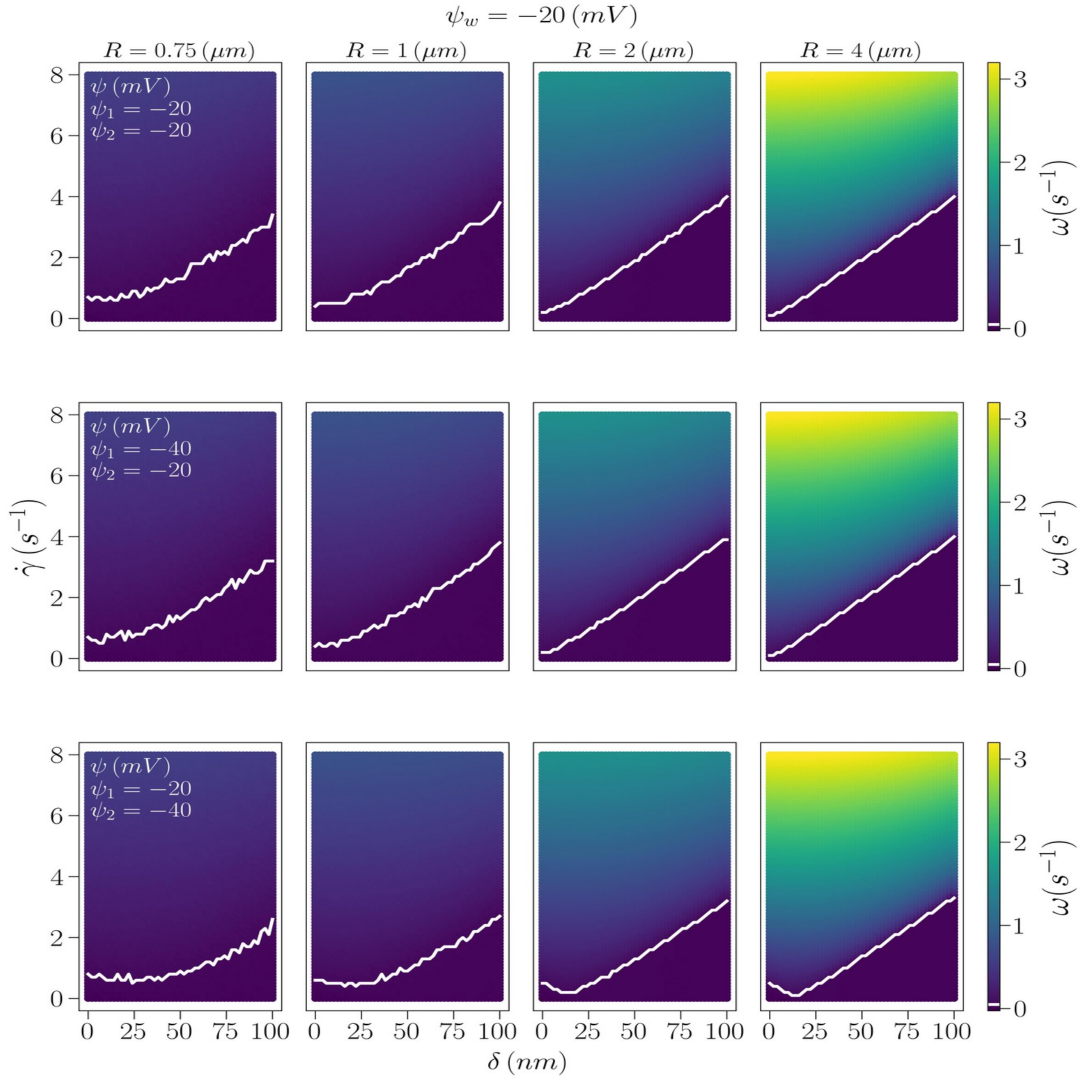

Figure S8: Angular velocity maps of a bottom-heavy Janus particle under varying shear flow. The wall charge is set to $\psi_w = -20\,mV$. From top to bottom, particle surface charges are $\psi_1 = -20\,mV$, $\psi_2 = -20\,mV$(top), $\psi_1 = -40\,mV$, $\psi_2 = -20\,mV$(middle), $\psi_1 = -20\,mV$, $\psi_2 = -40\,mV$(bottom). From left to right: $R = 0.75, 1.00, 2.00$, and $4.00\,\mu m$.

Figures S8 and S9 further support the analysis provided in Section 6 of the manuscript: Systems with $(\psi_1 = \psi_2) = (-20, -20)$, $(-40, -20)$ are characterized by a monotonic dependence of the white transition line on $\dot{\gamma}$ as δ grows. For the system with $(\psi_1, \psi_2) =$

$(-20, -40)$, instead, the minimum around $\delta = 16\,nm$ is systematically observed, regardless of ψ_w. For larger particles, the non-monotonicity is particularly visible as the data become noisier when R decreases. This, in turn, is caused by the small range of ω values, which makes the transition line more noisy, not only in the proximity of the minimum. Nonetheless, it can be seen that on reducing R the minimum persists, but is less sharp. Overall, the results across all systems confirm that the presence of the minimum is a consequence of the charge imbalance between the two faces of the Janus particle and, in particular, that it is observed only when the heavy side of the particle is overcharged.

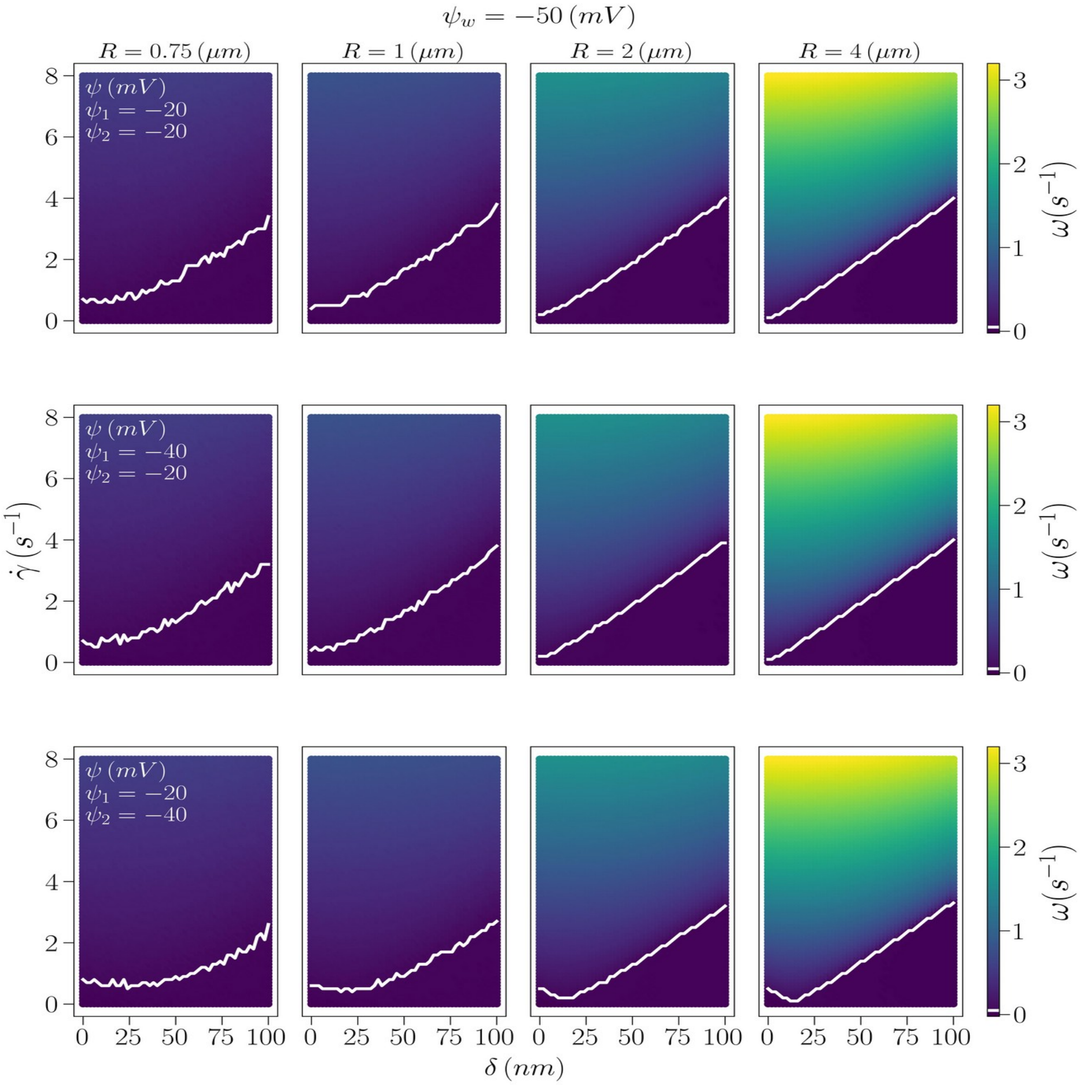

Figure S9: Angular velocity maps of a bottom-heavy Janus particle under varying shear flow. The wall charge is set to $\psi_w = -50\,mV$. From top to bottom, particle surface charge are $\psi_1 = -20\,mV$, $\psi_2 = -20\,mV$(top), $\psi_1 = -40\,mV$, $\psi_2 = -20\,mV$(middle), $\psi_1 = -20\,mV$, $\psi_2 = -40\,mV$(bottom). From left to right: $R = 0.75, 1.00, 2.00$, and $4.00\,\mu m$.

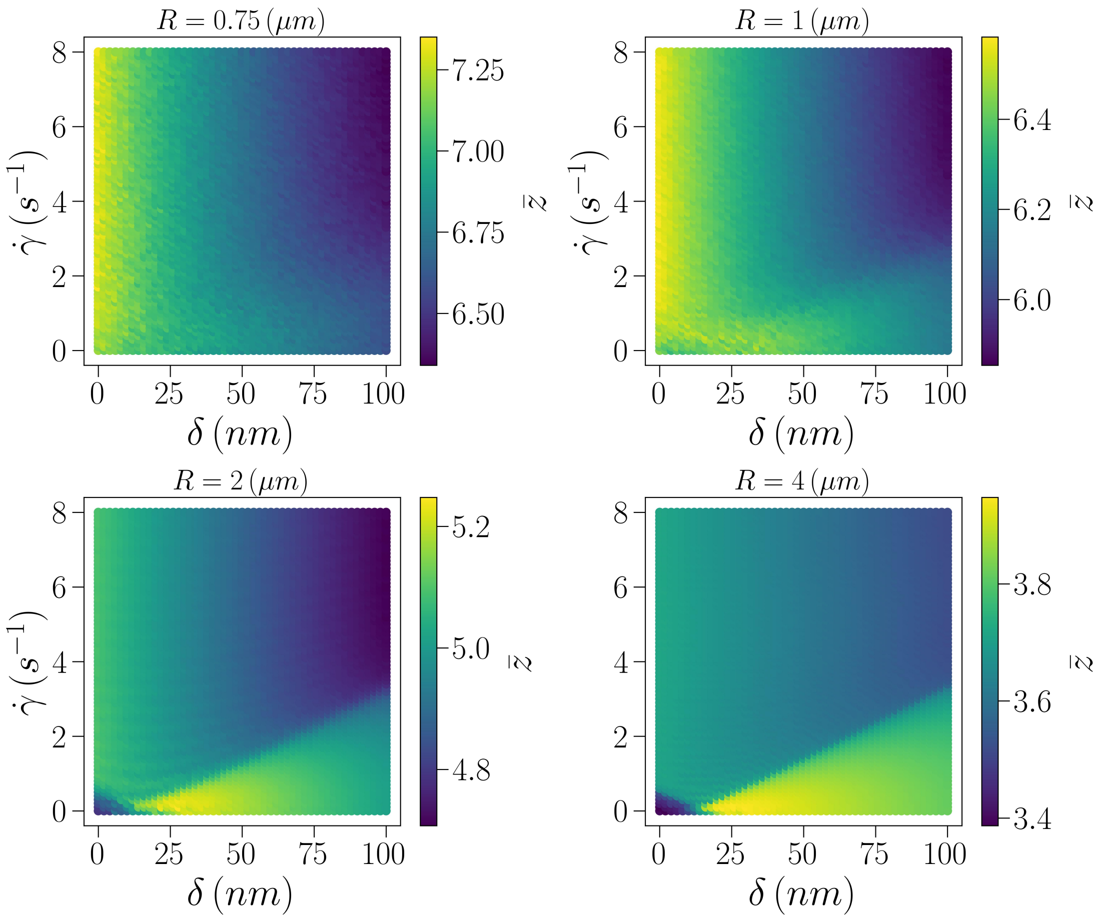

Figure S10: Average non-dimensionalized height, $\bar{z}$, as a function of cap thickness δ and shear rate, $\dot{\gamma}$, for bottom-heavy particles with radius $R = 0.75, 1, 2,$ and $4\,\mu m$. Surface charges are set to $\psi_1 = -20mV$, $\psi_2 = -40\,mV$ and $\psi_w = -50\,mV$.

Figure S10 shows the average non-dimensionalized height, $\bar{z}$, as a function of cap thickness, δ, and shear rate, $\dot{\gamma}$, for bottom-heavy particles with radius $R = 0.75, 1, 2,$ and $4\,\mu m$. Surface charges are set to $\psi_1 = -20\,mV$, $\psi_2 = -40\,mV$ and $\psi_w = -50\,mV$. The strong correlation between height and the behavior of θ seen in Figure S7 is also apparent in Figure S10, where the non-rotation region exists for all particle sizes except the smallest one. Note that particles with the smallest size considered in this study are also those that show the slowest rotation at given state points.

S6 State Diagrams for Model III

State diagrams derived using the analysis described in Section S2 are shown in Figures S11 and S12 for the same systems shown in Section S5. The transition line (white) between the rotating and non-rotating state is generally well-identified, with the entire non-rotating region classified as constant except for large particles with small cap thickness at small shear flow for which a relatively small region of unclassified state points

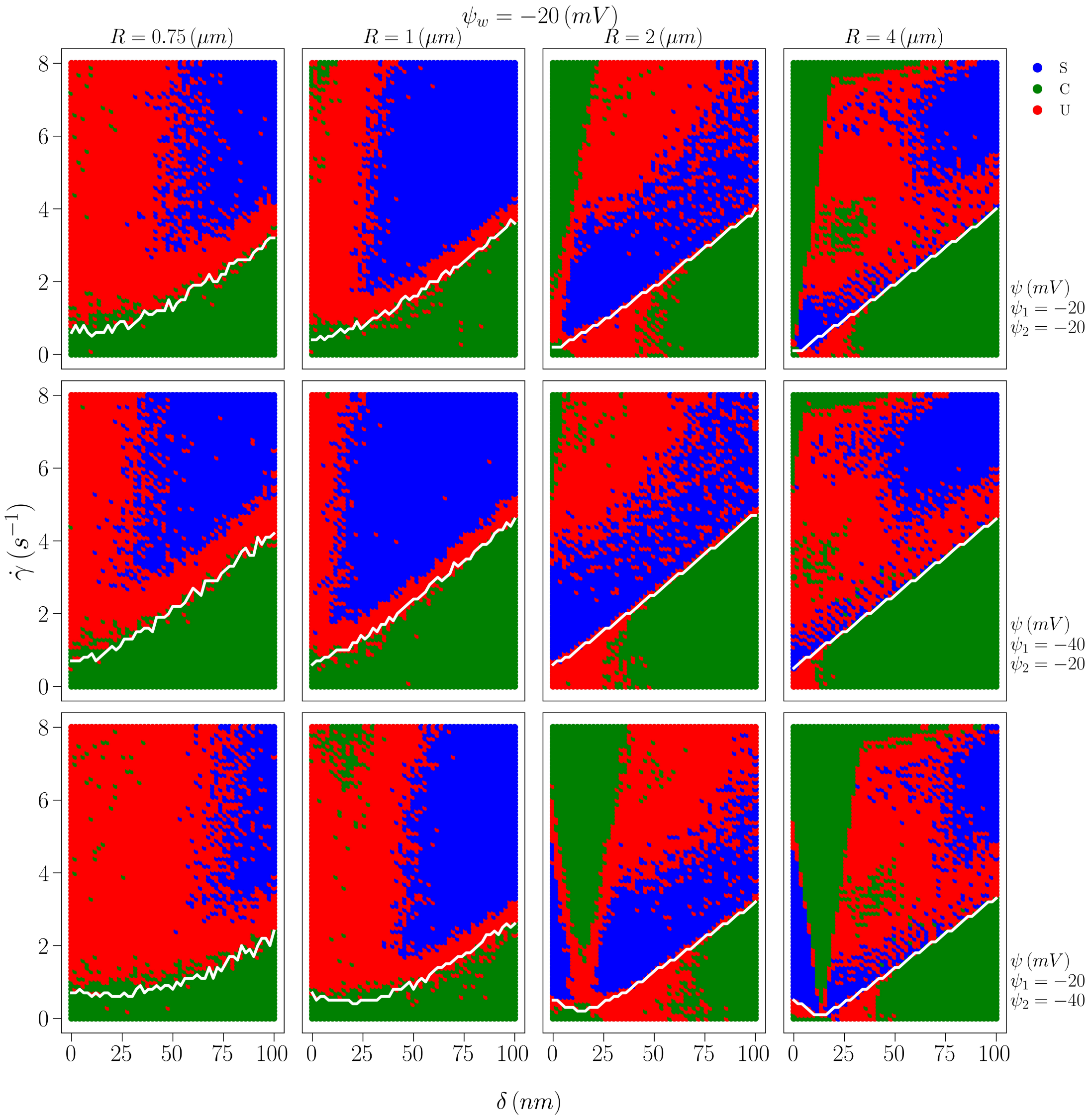

Figure S11: State diagrams of a bottom-heavy Janus particle under varying shear flow. The wall charge is set to $\psi_w = -20\,mV$. From top to bottom, particle surface charge are $\psi_1 = -20\,mV$, $\psi_2 = -20\,mV$(top), $\psi_1 = -40\,mV$, $\psi_2 = -20\,mV$(middle), $\psi_1 = -20\,mV$, $\psi_2 = -40\,mV$(bottom). From left to right: $R = 0.75, 1.00, 2.00$, and $4.00\,\mu m$.

is observed. As such regions are in the vicinity of the transition line, they are ascribed to the peculiar behavior of the system as exemplified by Figure 5 in the manuscript. In brief, at large δ and large $\dot{\gamma}$, there is a vast region of state points classified as sinusoidal, with the extension of the region weakly affected by the particle radius and affected by the charge imbalance. The surface charge value seems indeed to be the more relevant parameter in determining the transition from rotating states with constant angular velocity and the sliding behavior with time-varying angular velocity. Reducing δ implies to leave the region of state points classified as sinusoidal, i.e., the ones that are characterized by sliding behavior, and to enter a region of constant angular velocity. Such region has a

triangular shape and "moves" in the diagram with changing parameters consistent with the interpretation given in the manuscript.

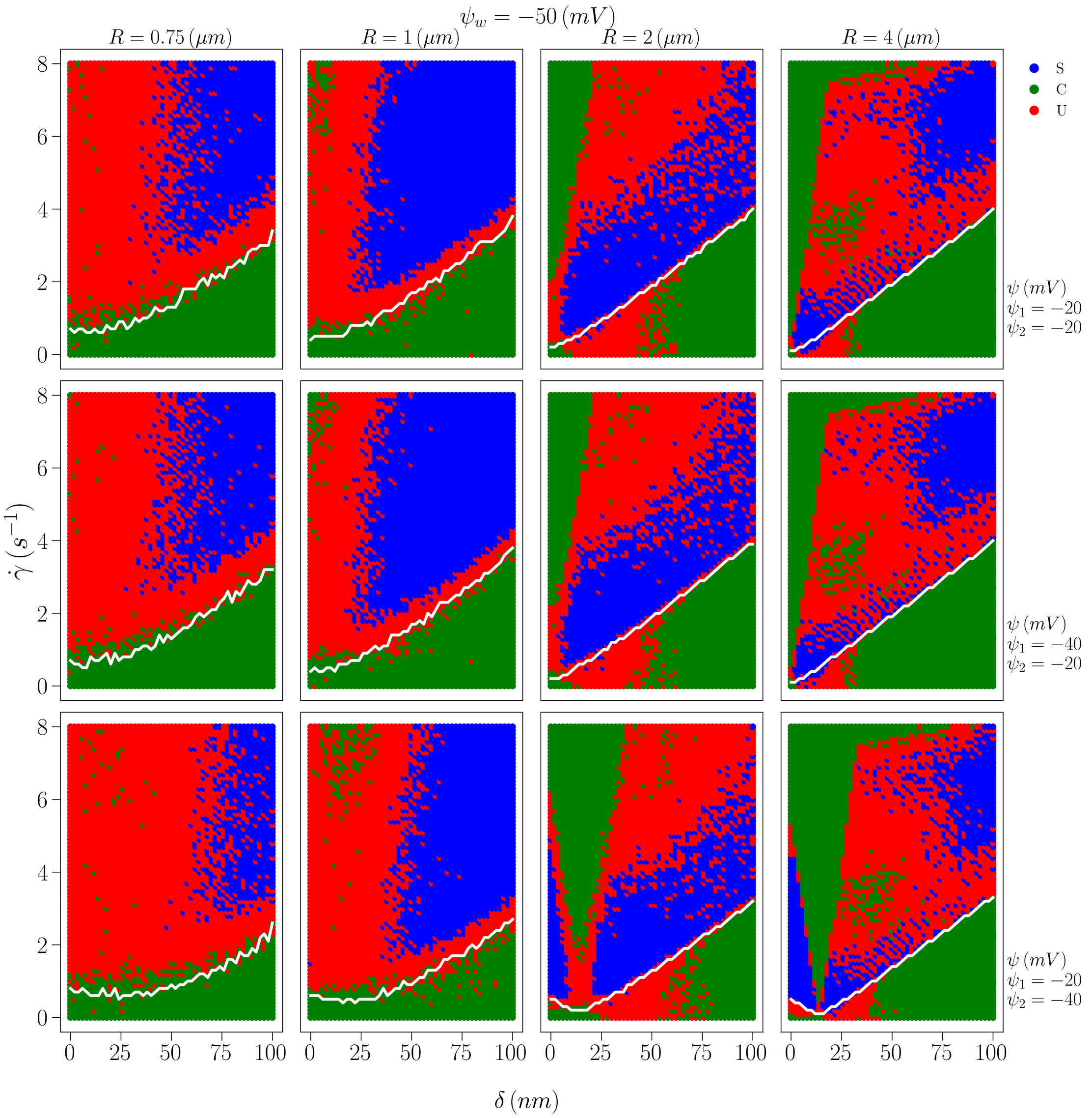

Figure S12: State diagrams of a bottom-heavy Janus particle under varying shear flow. The wall charge is set to $\psi_w = -50\,mV$. From top to bottom, particle surface charge are $\psi_1 = -20\,mV$, $\psi_2 = -20\,mV$(top), $\psi_1 = -40\,mV$, $\psi_2 = -20\,mV$(middle), $\psi_1 = -20\,mV$, $\psi_2 = -40\,mV$(bottom). From left to right: $R = 0.75, 1.00, 2.00$, and $4.00\,\mu m$.

S7 Impact of Hydrodynamic Friction in Model IV

The dynamical behavior of a bottom-heavy Janus particle using Models III and IV without (top two quadrants) and with friction (bottom two quadrants), respectively, is compared in Figure S13 for two particle sizes, $R = 1$ (left) and $R = 4\,\mu m$ (right). Each state

point is represented by three panels (from top to bottom): non-dimensionalized particle distance from origin along x-axis $\bar{x}$, particle orientation θ and non-dimensionalized particle height $\bar{z}$ shown as a function of non-dimensionalized time $\bar{t}$. Surface charges are set to $\psi_1 = -20\,mV$, $\psi_2 = -40\,mV$ and $\psi_w = -50\,mV$, while cap size is $\delta = 86\,nm$ and the shear rate is $\dot{\gamma} = 6.0\,s^{-1}$. Friction factors are $f_x = f_\theta = 1$ for the top two quadrants in

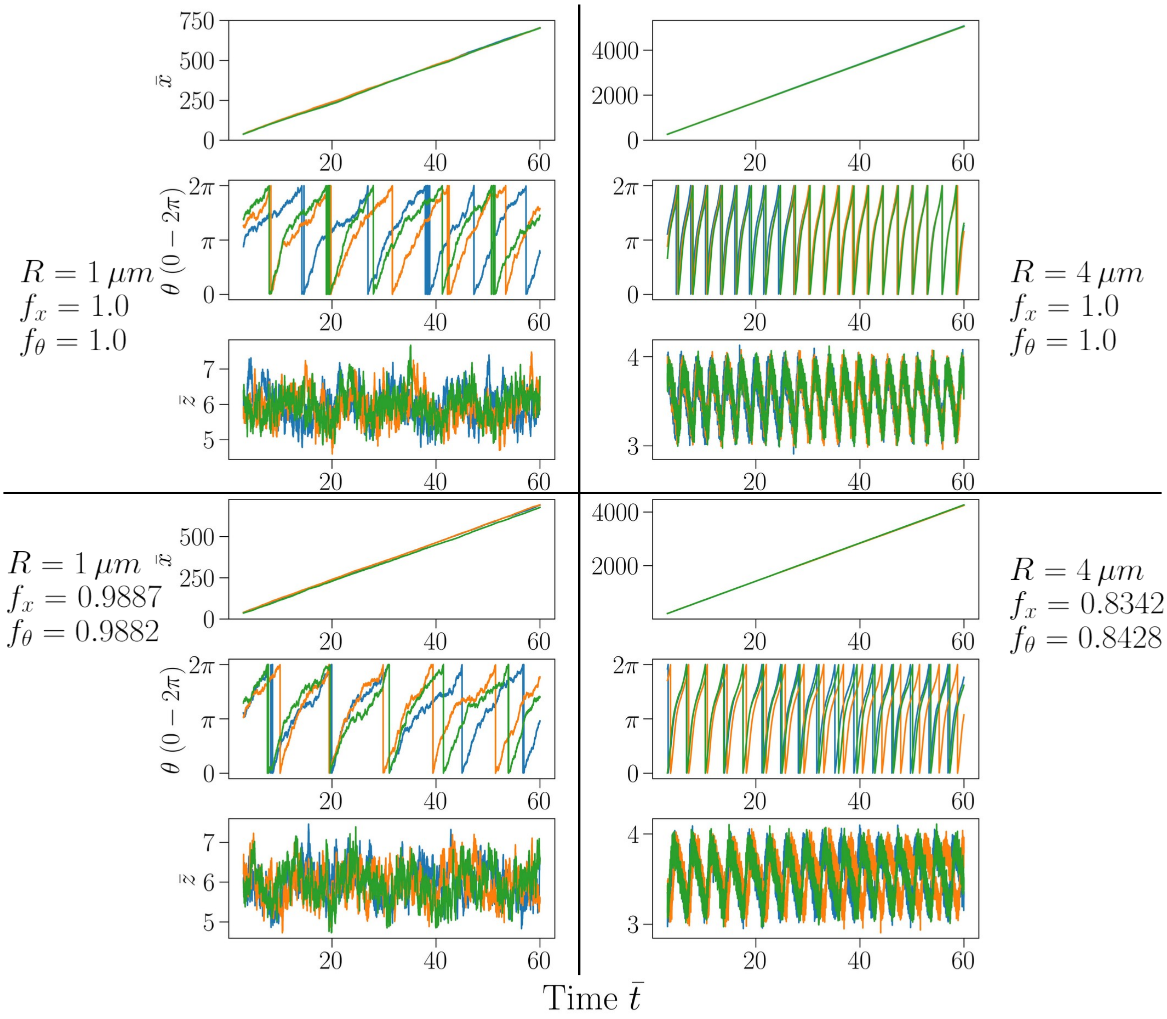

Figure S13: Impact of hydrodynamic friction - overall behavior of a bottom-heavy Janus particle in Model III (top) vs Model IV (bottom) for particle with $R = 1\,\mu m$ (left, $f_x = f_\theta = 1$ vs $f_x = 0.9887$ and $f_\theta = 0.9882$.) and $R = 4\ \mu m$ (right, $f_x = f_\theta = 1$ vs. $f_x = 0.8342$ and $f_\theta = 0.8428$). Surface charges are set to $\psi_1 = -20\,mV$, $\psi_2 = -40\,mV$ and $\psi_w = -50\,mV$, and $\delta = 86\,nm$ and $\dot{\gamma} = 6.0\,s^{-1}$. Three panels for each state from top to bottom are: non-dimensionalized particle distance from origin along x-axis $\bar{x}$, particle orientation θ and non-dimensionalized particle height $\bar{z}$ shown as a function of non-dimensionalized time $\bar{t}$.

the absence of hydrodynamic friction. In the presence of hydrodynamic friction, friction factors based on Goldman et al.[5] of $f_x = 0.9887$ and $f_\theta = 0.9882$ are used for $R = 1\ \mu m$ (bottom left quadrant) and $f_x = 0.8342$, $f_\theta = 0.8428$ for $R = 4\ \mu m$ (bottom right quadrant).

Comparing each top panel with the corresponding lower panel shows that hydrodynamic friction has virtually no impact on the $R = 1\,\mu m$ particle behavior beyond reducing the maximum distance traveled, which diminishes when hydrodynamic friction is accounted for. In the $R = 4\,\mu m$ particle case, the particle also travels less far, but additionally experiences fewer rotations due to hydrodynamic friction, which is expected since it is closer to the wall due to its larger size.

S8 Impact of EDL torque, N_{EDL}, in Model III

The weighting factors, g_+ and g_-, introduced in Eq. 4 in the manuscript, allow contributions from the entire particle surface to the EDL interaction, where interactions from the face pointing away from the wall contribute significantly less. Figure S14 shows the U_{EDL} potential (left) and the resulting torque, N_{EDL}, (rigth) as a function of particle orientation in radians for a SiO_2 particle located at $\bar{z} = 6$ above a wall with potential $\psi_w = -50$ mV surrounded by water of ionic strength $I = 1 \cdot 10^{-6}$ M at 300 K with particle surface potentials of $\psi_1 = -20$ and $\psi_2 = -40$ mV.

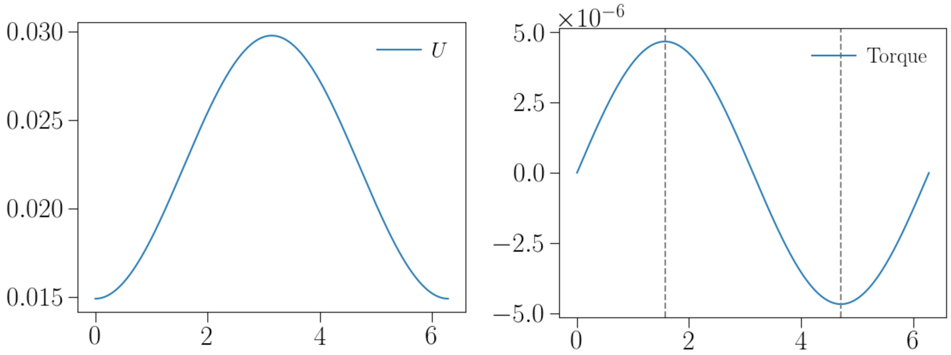

Figure S14: U_{EDL} and resulting N_{EDL} when weighting factors, g_+ and g_-, are used.

Rashidi et al.[6] showed in their work that at high ionic strength, only the particle surface that is within $\pm(10-15)^\circ$ of the wall surface normal contributes to the EDL interaction, i.e., the Janus nature of the particle only becomes important when the Janus boundary is within $\pm(10-15)^\circ$ of the wall surface normal. To test the impact of the EDL torque resulting from the use of the weighting factors, g_+ and g_-, two N_{EDL} torque functions, N^3_{EDL} and N^{31}_{EDL}, are constructed that artificially reduce the range within which the EDL torque acts on the particle to the range observed by Rashidi et al.[6] and one in between. The shape of the original and the two modified torque functions is shown in Fig. S15, where all curves have been normalized to have a unitary maximum. In N^{31}_{EDL} the torque function is zero everywhere except when Janus boundary points toward the wall within 15° (dashed lines in Fig. S15). The functions are obtained by raising the original torque N_{EDL} to an odd power. As this mathematical operation strongly affects the maximum that the torque functions have, i.e., the intensity of the torque, the torque functions are normalized such that their maximum represents the absolute value of the original torque at each height $\bar{z}$ (and for each value of the radius R and all surface potentials).

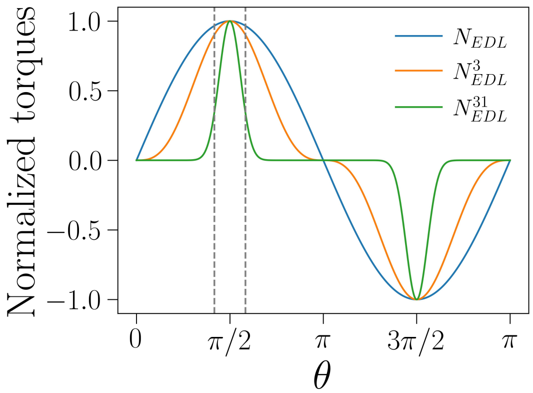

Figure S15: Original and modified torque functions that are zero everywhere except when the Janus boundary is close to the wall normalized to have unitary maximum.

State Diagrams obtained with N_{EDL}, N^3_{EDL}, and N^{31}_{EDL} are shown in Fig. S16. Note that Figure S16A is obtained under the same conditions as Figure 6C in the manuscript.

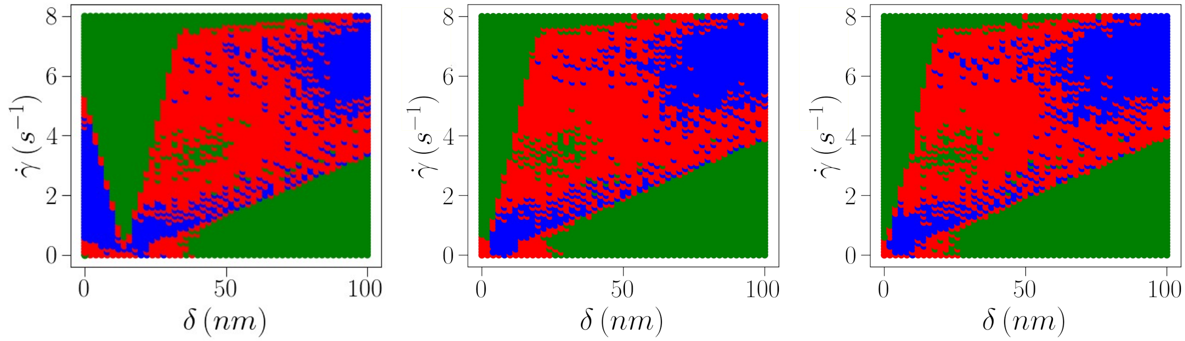

Figure S16: State diagram for the model with $R = 4$ μm, $\psi_1 = -20$ mV, $\psi_2 = -40$ mV and $\psi_w = -50$ mV. From left to right, data have been obtained by raising the N_{EDL} torque to the power of 1 (the original data, blue curve in Fig. S15), to the power of 3 (orange curve in Fig. S15) and to the power of 31 (green curve in Fig. S15)

The main change observed in the state diagrams obtained with N^3_{EDL} and N^{31}_{EDL} is a left shift of the position of the down-pointing green triangle (region dominated by constant, non-zero, angular velocity) for thin cap values. Interestingly, the shift is already observed for very small changes in the torque, N^3_{EDL}, and no additional changes are seen when a much narrower torque is used, N^{31}_{EDL}. We believe that this observation confirms that as a first simplified approximation the weighting factors, $g_\pm(\theta) = \frac{(1\pm\cos\theta)}{2}$, can be used. In addition, the disappearance of sinusoidal region (blue) at low shear rates for thin caps may provide an experimental route to test the accuracy of the weighting factors.

References

[1] G. Trefalt, S. H. Behrens and M. Borkovec, *Langmuir*, 2016, **32**, 380–400.

[2] S. Pelargonio and A. Zaccone, *Phys. Rev. E*, 2023, **107**, 064102.

[3] K. P. Burnham and D. R. Anderson, *Model Selection and Multimodel Inference: A Practical Information-Theoretic Approach*, Springer, New York, 2nd edn, 2002.

[4] G. Volpe, T. Brettschneider, L. Helden and C. Bechinger, *Opt. Express*, 2009, **17**, 23975–23985.

[5] A. Goldman, R. Cox and H. Brenner, *Chemical engineering science*, 1967, **22**, 653–660.

[6] A. Rashidi and C. L. Wirth, *The Journal of Chemical Physics*, 2017, **147**, 224906.
